# Nb-Doped VO_2_‑Based Coatings on Glass: Substrate Effects, Thermochromic Performance, and an Effective Transition Temperature for Smart-Glazing Applications

**DOI:** 10.1021/acsami.6c03088

**Published:** 2026-06-12

**Authors:** Antonio J. Santos, Andrea Casas-Acuña, José M. Mánuel, Juan J. Jiménez, Jose M. Obrero-Pérez, Davide Benedetto, Nicolas Martin, Francisco M. Morales

**Affiliations:** † IMEYMAT: Institute of Research on Electron Microscopy and Materials of the University of Cádiz, Puerto Real E-11510, Spain; ‡ Department of Materials Science and Metallurgic Engineering, and Inorganic Chemistry, Faculty of Sciences, University of Cádiz, Puerto Real E-11510, Spain; § Department of Condensed Matter Physics, Faculty of Sciences, University of Cádiz, Puerto Real, Cádiz 11510, Spain; ∥ SUPMICROTECH, 88524 CNRS, Institut FEMTO-ST, Besançon, Cedex 25000, France

**Keywords:** smart glazing, vanadium dioxide, Nb-doping, glancing angle codeposition, rapid thermal annealing, substrate effect, thermochromic behavior

## Abstract

Niobium-doped VO_2_ thin films were deposited on soda-lime and borosilicate glass substrates to investigate the coupled effects of dopant concentration, substrate chemistry, and thermal processing on microstructure, thermochromic performance, and metal–insulator transition (MIT) kinetics. Coatings with nominal Nb contents of 3, 4.5, and 6 at. % were fabricated using controlled mono- and multilayer codeposition strategies followed by rapid thermal annealing. Structural and morphological analyses confirm VO_2_(M) as the dominant phase on both substrates, while revealing a strong substrate-dependent stability of secondary phases driven by Na diffusion, especially in soda-lime glass. Advanced scanning-transmission electron microscopy image and spectroscopic-related techniques demonstrate that Nb exhibits a less homogeneous spatial distribution than other dopants (such as W) under comparable processing conditions, leading to compositional modulations and multistep MIT behavior, particularly on soda-lime substrates. In contrast, borosilicate glass enables enhanced grain coalescence, reduced Na-induced phase instability, and a more uniform Nb incorporation. These microstructural differences directly impact the optical response. Borosilicate-supported coatings exhibit a more favorable balance between luminous transmittance (*T*_lum_) and solar modulation efficiency (Δ*T*_sol_), while films on soda-lime glass show a progressive degradation of Δ*T*_sol_ at higher Nb contents. Despite a lower *T*_c_ reduction efficiency compared to W, Nb doping preserves or even improves *T*_lum_ at elevated concentrations. Finally, an application-oriented effective transition temperature, *T*_eff_, is proposed as a more realistic metric for assessing the suitability of VO_2_-based coatings for smart-glazing applications.

## Graphical abstract

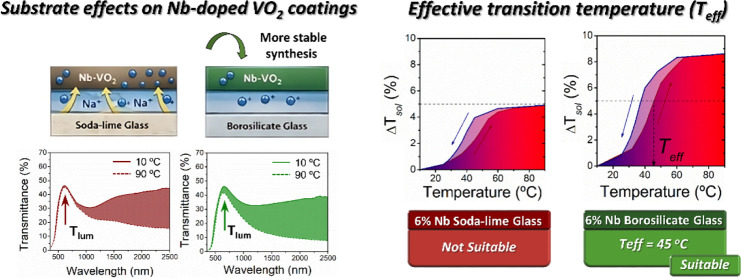

## 1 Introduction

The increasing demand for energy-efficient technologies has made the optimization of building envelopes, particularly glazing systems, a key research priority.^1–5^ In recent years, extensive efforts have been devoted to developing energy-efficient materials for smart glazing in response to the growing demand for energy savings.^6–10^ Thermochromic materials, which regulate solar energy transmission as a function of temperature, have emerged as particularly suitable candidates for this purpose.^8,11–13^ Among these, vanadium dioxide (VO_2_) stands out because it undergoes a reversible metal–insulator transition (MIT) associated with a structural phase transformation at a critical temperature (*T*_c_ ≈ 68 °C).^12,14–16^ This switchable optical behavior enables a VO_2_-based coating to maximize solar heat gain under cold conditions (monoclinic phase) while effectively mitigating overheating in warm conditions (tetragonal, rutile phase).^1,17^

Despite these advantages, the practical implementation of VO_2_ in smart windows remains hampered by several intrinsic limitations, including its relatively high-phase transition temperature, low luminous transmittance (*T*_lum_), and modest solar modulation ability (Δ*T*_sol_).^5,18–22^ Numerous strategies have been explored to overcome these issues. Among these strategies, chemical doping has emerged as one of the most effective routes to tailor the transition temperature. However, modifying the MIT often leads to trade-offs in optical and thermochromic performance, complicating the optimization of VO_2_-based coatings.^8,23^

Phase transition temperature reduction is typically achieved by incorporating dopant cations with larger ionic radius than V^4+^.^24^ Tungsten is widely regarded as the most efficient dopant, lowering *T*_c_ by ∼20–26 °C per W atom percentage unit;^13,25^ however, W doping often degrades optical properties,^21,26^ restricting its applicability in smart windows. By contrast, Nb doping has been reported to improve *T*_lum_ without excessively compromising Δ*T*_sol_.^21,27^ Nevertheless, compared to W, the effects of Nb incorporation remain significantly less explored, particularly regarding dopant distribution, phase stability, and MIT kinetics in VO_2_ thin films.

VO_2_ thin films have been synthesized by various techniques, including chemical vapor deposition, pulsed laser deposition, solution-based methods, polymer-assisted deposition, sol–gel processing, and magnetron sputtering.^24,28,29^ Among these, sputtering stands out due to its scalability and flexibility to control the film architecture and dopant incorporation.

Moreover, the choice of the substrate plays a critical role in determining the structural and functional properties of VO_2_ coatings. In particular, alkali diffusion from widely used soda-lime glass can strongly affect phase stability and microstructure, leading to substrate-dependent performance variations.^30^ However, the interplay between the substrate chemistry and Nb incorporation has not been systematically addressed.

To address these gaps, the present work investigates the fabrication of Nb-doped VO_2_-based thin films deposited on two types of technologically relevant glass substrates: commercial soda-lime and borosilicate glasses. Different doping strategies based on single-layer and multilayer architectures are employed to control dopant incorporation, followed by rapid thermal annealing to stabilize the VO_2_(M) phase. This study systematically analyzes the coupled effects of dopant concentration, film architecture, processing conditions, and substrate chemistry on microstructure, phase stability, and thermochromic performance with particular emphasis on dopant distribution and MIT characteristics relevant for smart-glazing applications. Finally, an application-oriented effective transition temperature (*T*_eff_) is introduced as a more representative metric for assessing the practical performance of smart coatings in real-world environments.

## 2 Materials and Methods

### 2.1 Deposition Process

Thin films were deposited at room temperature by direct current (DC) magnetron sputtering using two opposite metallic targets (51 mm of diameter and 99.9% purity): vanadium and niobium. They were located inside a 40 L homemade vacuum chamber evacuated down to 10^–5^ Pa before each run by means of a turbomolecular pump backed by a primary pump. The distance between V and Nb target centers and the glass substrates (Menzel Gläser microscope slides and Borofloat) were fixed to 65 mm and 95 mm, respectively. Following the procedure described in our previous studies,^31–34^ porous films with large surface-to-volume ratios and enhanced reactivity with oxygen were deposited by combining GLAD and reactive gas pulsing process (RGPP) techniques. The deposition angles α (average angle of the incoming particle flux) relative to the substrate normal were set at α = 85° and 75° for vanadium and niobium, respectively, with no rotation of the substrate (i.e., ϕ = 0 rev h^–1^). Argon was injected at a mass flow rate of 2.40 sccm, and the pumping speed was maintained at *S* = 13.5 L s^–1^, whereas the oxygen gas was periodically supplied into the sputtering chamber. A rectangular pulsed signal was employed for the oxygen flow rate with respect to time evolution. The pulsing period was set at *P* = 16 s. The maximum oxygen flow rate was *q*_O2Max_ = 0.40 sccm. It corresponds to the critical flow required to trigger the process in the compound sputtering mode. The minimum oxygen flow rate was *q*_O2Min_ = 0 sccm, while the oxygen injection time (*t*_ON_) was set at 8 s. Under this premise, V_*x*_Nb_1–*x*_O_*y*_ layers of different compositions and configurations were achieved by adjusting the deposition times of each element for fixed vanadium and niobium target currents of 200 and 20 mA (minimum current to generate a stable Nb plasma), respectively. A nominal film thickness of 80 nm was achieved by fixing the total deposition time according to an average deposition rate of 240 nm h^–1^, which was previously determined for the deposition of vanadium metal at α = 85°. Finally, samples were subjected to an azimuthal rotation of Φ = 180° at the halfway point of the deposition time for each layer. This procedure promoted the fabrication of zigzag GLAD films with greater thickness homogeneity. This, in turn, simultaneously ensured a more balanced porosity gradient by effectively compensating for preferred shadowing effects.

### 2.2 Thermal Treatments

Oxidation was performed at temperatures ranging from 475 to 550 °C using an RPT-100 rapid thermal annealing (RTA) system (UniTemp GmbH). This system features 18 infrared 20 kW lamps and a graphite susceptor to ensure a uniform reaction surface for substrates up to 100 mm diameter (4″) or 100 mm × 100 mm^2^. A constant internal airflow of 1 lpm (liter per minute) of compressed air was maintained during the thermal treatments. The RTA process involved rapid heating ramps (40 °C s^–1^). The air overpressure, finely stabilized by a mass flow controller, prevents the local depletion of O_2_ that can occur in static or open-tube oxidative atmospheres. The selection of the corresponding holding times at each temperature was guided by the well-established temperature-reaction kinetics relationship governing oxidation processes, whereby higher annealing temperatures require shorter reaction times to achieve comparable oxidation levels. Accordingly, holding times were systematically adjusted as a function of temperature and further optimized for each substrate in order to maximize the formation of the VO_2_(M) phase while minimizing secondary phases, defining substrate-dependent processing windows. Eventually, postannealing cooling was carried out at an approximate rate of –10 °C s^–1^ down to ∼200 °C, achieved by maintaining a constant airflow of 25 lpm within the reaction chamber.

### 2.3 Structural, Compositional, and Functional Characterizations

Different structural and spectroscopic techniques associated with electron microscopy were applied to local areas (i.e., from ∼100 nm^2^ to a few μm^2^) to characterize the structure and composition of the fabricated materials. In this context, 3 different ThermoFisher Scientific electron microscopes were utilized, at the facilities of the Central Scientific and Technological Research Services (University of Cádiz): a NanoSEM 450, a Scios2 DualBeam with a focused ion beam (FIB) module and a TALOS F200X STEM microscope. The NanoSEM and Scios2, both scanning electron microscopes (SEM), were utilized to obtain secondary electron images from the material surface at low voltage (5 kV) with the Everhart–Thornley detector (SEM-ETD). This allowed us to compare the differences in microstructure and thicknesses of the fabricated materials within micrometric fields of view. Besides, the FIB module in the Scios2 was also utilized to perform perpendicular trenches at the thin film surfaces, useful to study the films’ depth profiles (cross sections) and to prepare electron-transparent lamellas (in cross-sectional orientations) that were subsequently studied via transmission and scanning-transmission electron microscopy, (S)­TEM using the TALOS microscope, working at 200 kV. The applied (S)­TEM-related techniques, for structural and chemical characterization at the nanometer level, included bright-field-diffraction contrast TEM (BFTEM) and high-resolution TEM (HRTEM) imaging, using a 40 mm objective aperture; high-angle annular dark-field imaging (HAADF) and spectroscopies such as electron energy-loss spectroscopy (EELS) and energy-dispersive X-ray spectroscopy (EDX). A Gatan Imaging Filter (GIF) Continuum system fitted in the TALOS microscope was used for spatially resolved EELS analysis in STEM mode. STEM-EELS 2D spectrum image (SI) data were acquired using a 2.5 mm diameter aperture and a 0.05 eV/channel energy dispersion. The camera length was 47 mm, so the convergence and collection semiangles were thus set to 10.5 and 20.0 mrad, respectively, and the probe current was about 150–175 pA depending on the experiment. In order to allow accurate chemical shift measurements, the dual EELS mode was used to record nearly simultaneously both low-loss signal and the V-L_2,3_ and O–K high-loss edges, at each pixel position. A dwell time of about 0.2 spp (second per pixel) was set to optimize the signal-to-noise ratio. EDX point spectra and 2D spectroscopic maps were recorded using a Super-X detection system that is constituted by four silicon drift detectors distributed around the electron-transparent sample inside the microscope. Eventually, EDX spectra were also recorded along 30 kV SEM images, using a 100 mm^2^-surface EDAX detector (AMETEK), coupled to the Nova NanoSEM450 microscope. All the spectrum results in this work refer to the net signal, after removing the background. In this sense, a power law model between the intensity and the energy loss has been considered in order to remove the background (due to the zero-loss peak) for EELS spectra. GIXRD scans were performed on a Malvern Panalytical Aeris diffractometer (Cu radiation) working at 30 kV (10 mA) and setting a grazing incidence angle of 0.8°. The scanning step size and time per step were set to 0.01° and 0.264 s, respectively.

XPS analyses were performed with a Kratos Axis Ultra DLD spectrometer (Kratos Analytical Ltd., Manchester, UK), using a monochromatic Al Kα source (*h*ν = 1486.6 eV). The operating power was 150 W, and the vacuum condition in the analysis chamber was 2 × 10^–9^ Torr. Survey and high-resolution spectra were acquired with a step energy of 80 and 20 eV, respectively, in the fixed analyzer transmission mode. Samples were mounted on a stainless-steel holder using a double-sided conductive carbon tape. O 1s signal at 530.0 eV was used to calibrate the binding energies. High-resolution spectra of individual elements (Na 1s, O 1s, V 2p, and Nb 3d) were collected with 40 accumulations to improve the signal-to-noise ratio. For depth profile analysis, the samples were subjected to Ar^+^ ion etching (10^–6^ mbar, 20 kV, 240 min). The incidence angle between the ion beam and the sample surface was 130°, and the etched area was 4 × 4 mm^2^.

The thermochromic optical features of the fabricated Nb-doped VO_2_ films were determined using a Cary 5000 UV–vis–NIR spectrophotometer (Agilent Technologies). The system was equipped with a Microptik MHCS120-XY stage for precise temperature control. Transmittance spectra were recorded in the wavelength range of 300–2500 nm at selected temperatures in the range of 10–90 °C. For the dynamic monitoring of the thermally induced phase transition, the thermal evolution of the optical transmittance at a selected NIR wavelength (2500 nm) was recorded during consecutive heating–cooling cycles at a controlled rate of 5 °C min^–1^.

## 3 Results and Discussion

### 3.1 Calibration of Nb Doping and Definition of Sample Architectures

Previously to the coating fabrication, it was first necessary to adjust the codeposition conditions to achieve the desired doping ratios. For this purpose, a reference sample of V_*x*_Nb_1–*x*_O_*y*_ with a nominal thickness of 300 nm was deposited under the continuous target current conditions established in the previous section. It is worth noting that, at this stage, the zigzag deposition was omitted to simplify and expedite the manufacturing processes. Likewise, a simplified nomenclature was adopted to designate the samples, considering that after thermal treatments, the Nb/V ratios of the V_*x*_Nb_1–*x*_O_*y*_ precursors are ideally preserved in the formed V_*x*_Nb_1–*x*_O_2_. Thus, samples with (1 – *x*)/*x* ratios will be designated as (1 – *x*) Nb; for example, Nb/V = 3/97 will be denoted as 3Nb. The cross-sectional image and SEM–EDX analyses (standardless quantification) of this reference sample are shown in Figure 1a,b respectively, revealing a Nb/(V + Nb) atomic content of ∼6 at. %. However, it has been reported in the literature that the concentrations at which niobium functions effectively as a dopant in VO_2_(M)-based smart glasses, i.e., without significantly impairing luminous transmittance or infrared modulation efficiency, are typically below 7Nb.^27,35^ In the case of this work, EDX maps have been obtained for samples 4.5Nb_GL_2, 4.5Nb_BF_1, 6Nb_GL_1, and 6Nb_BF_1 (see detailed compositional measurements in Table S3, at the Supporting Information, Section II), having obtained an atomic percentage for Nb of (1 – *x*) equal to 2.66 ± 0.43, 2.81 ± 0.45, 3.99 ± 0.53, and 4.08 ± 0.54, respectively. Thus, the actual Nb atomic content is assumed to be slightly lower than the nominal one, though the trend among the samples (increasing Nb content from 3Nb- to 6Nb-samples) is kept, which is key for the discussions in this work.

**Figure 1:**
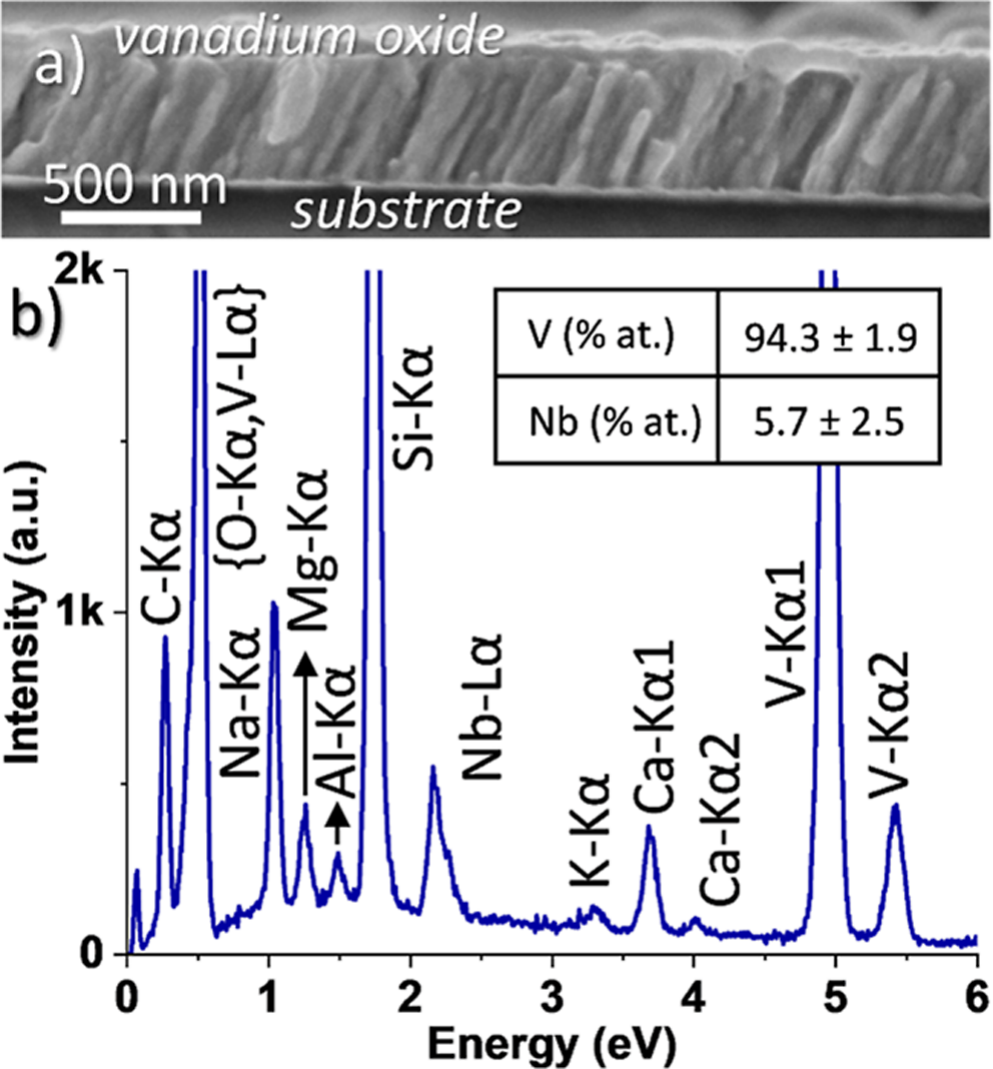
(a) Cross-sectional SEM image of the V_0.94_Nb_0.06_O_*y*_ reference sample and (b) integrated EDX spectrum acquired from the film region observed in the SEM image.

To reach nominal Nb contents below 6Nb, two complementary deposition strategies were employed. The first consisted of fabricating alternating VO_*y*_/V_0.94_Nb_0.06_O_*y*_ multilayers, following the methodology previously reported.^32^ This approach allows lowering the overall dopant level by controlling the number and thickness of Nb-containing sublayers through timed opening and closing of the Nb shutter. The second strategy involved the deposition of a single-layer 6Nb film, corresponding to the minimum dopant concentration obtainable when both targets operate under continuous flux. These two architectures (multilayers for <6Nb and single layer for 6Nb) do not only enable the control of global composition but also provide a means to assess whether multilayer designs introduce additional interfacial barriers that could hinder the homogeneous diffusion and incorporation of Nb into the VO_2_(M) lattice during subsequent annealing. Regarding the multilayer structure, the total dopant concentration was adjusted by modifying the deposition time of each V_0.94_Nb_0.06_O_*y*_ sublayer. A total of 12 equidistant Nb-containing sublayers was selected for compositions below 6Nb, maximizing both the number of diffusion fronts and the effective randomization of Nb during thermal treatment.

Once these parameters were defined, 80 nm thick zigzag-GLAD Nb-containing coatings were deposited on both glass substrates (soda-lime and borosilicate). A slightly thicker film than in previous studies was intentionally selected to enhance the observability of Nb-induced trends not only in the transition temperature but also in *T*_lum_ and Δ*T*_sol_, which become more distinguishable for thicker layers. A series of V_*x*_Nb_1–*x*_O_*y*_ films with nominal Nb contents of 3Nb, 4.5Nb, and 6Nb were fabricated. Each of these coatings was then subjected to different RTA treatments in order to identify optimal oxidation conditions as a function of substrate, composition, and layer architecture, obtaining a total of 24 samples. Among these, the 10 most optimal samples, selected based on their functional performance, are listed in Table 1, together with their corresponding deposition and annealing parameters. It should be noted that the term “optimal” in this context refers specifically to the processing window that maximizes VO_2_(M) phase purity and thermochromic response for the specific Nb-doping levels and glass substrates explored in this work. For the intermediate composition (4.5Nb), and for both substrates, 3 annealing temperatures (475, 525, and 550 °C) were included in this optimal subset to examine the specific influence of oxidation temperature. The remaining 14 samples, together with their optical/thermochromic properties, are compiled in the Supporting Information file, Section I.

**Table 1:** Collection of Samples Fabricated and Characterized in This Work, Indicating Their Labeling and Distinctive Fabrication Parameters^a^

Label	Nb atomic doping in V_*x*_Nb_1–*x*_O_2_	Thickness ratio between V_0.94_Nb_0.06_O_*y*_ and VO_*y*_	Substrate	*T*_r_ (°C)	*t*_r_ (s)
3Nb_GL_1	1.00	40/40	soda-lime	525	12
3Nb_BF_1			borosilicate	475	90
4.5Nb_GL_1	1.50	60/20	soda-lime	475	75
4.5Nb_GL_2				525	10
4.5Nb_GL_3				550	5
4.5Nb_BF_1			borosilicate	475	90
4.5Nb_BF_2				525	25
4.5Nb_BF_3				550	10
6Nb_GL_1	2.00	80/0	soda-lime	525	5
6Nb_BF_1			borosilicate	475	120

a *T*_r_ and *t*_r_ are the thermal annealing temperature (°C) and time (s), respectively.

It is important to clarify that the nomenclature adopted throughout this work refers solely to the nominal Nb content introduced during deposition and should not be interpreted as the amount of Nb that is effectively incorporated into the VO_2_(M) lattice. In multilayer architectures, Nb atoms must diffuse across sublayers during annealing, a process that may lead to partial local aggregation, rather than uniform incorporation. Therefore, the indicated composition (e.g., 3Nb, 4.5Nb, or even 6Nb) should be understood as the intended overall Nb/V ratio established during deposition, instead of the final, spatially homogeneous Nb distribution within the oxidized film.

### 3.2 Morphological, Structural, and Compositional Characterization

Figure 2 gathers SEM–ETD micrographs from the surface and cross-section (FIB trench-prepared) of the optimal sample subset, as indicated in the previous section. However, for the cross-sectional views of the 6Nb samples, electron-transparent FIB lamellae were prepared, and low-magnification HAADF images are presented instead. Surface cross-sectional view images in this figure are presented at the same magnifications; thus, one scale is given for all images of each type. In most of the cases, cross sections exhibit an apparently granular and relatively compact morphology on both substrate types. Vanadium oxide film thicknesses have been measured from cross-sectional views in Figure 2, and the results are presented in Table 2. The average layer thicknesses are around 135 nm for coatings on soda-lime glass and 145 nm for those on borosilicate substrates. These values are noticeably larger than the nominal deposition thickness, an effect commonly observed after thermal annealing as a consequence of oxygen uptake and film oxide densification.^36^ Importantly, no significant differences or clear trends in the overall thickness are detected between samples fabricated on the two substrates.

**Figure 2:**
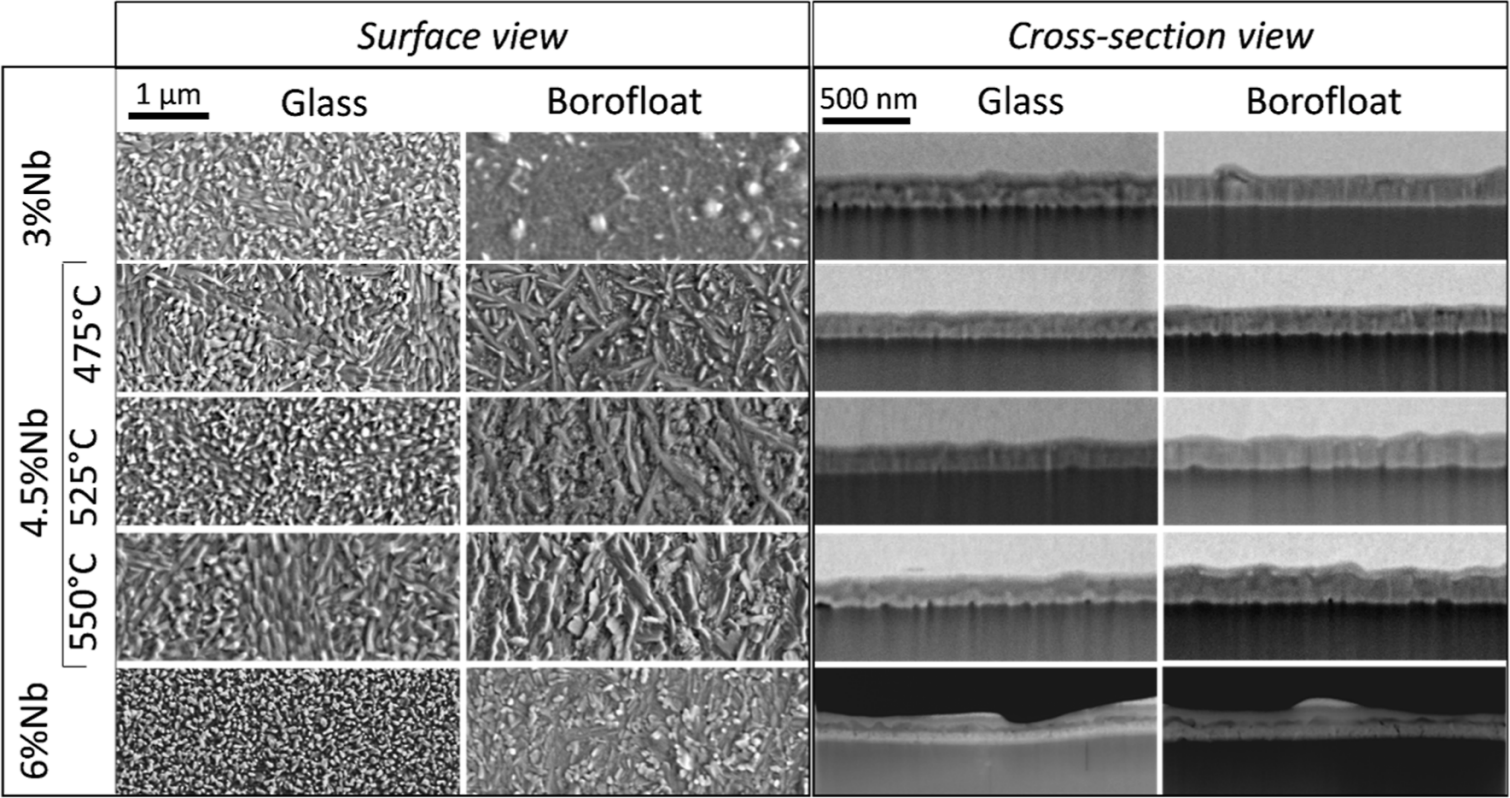
SEM micrographs (top view and cross section) for representative compositions and substrates. Cross-sectional views, in the case of samples 6Nb, correspond to STEM-HAADF images from electron-transparent FIB lamellas.

**Table 2:** SEM Measured Average Layer Thicknesses for the Optimal Sample Selection Extracted from SEM Cross Sections

Sample	Layer thickness (nm)	Grain size (nm)	Surface feature size (nm)
3Nb_GL_1	147 ± 15	228 ± 48	-
3Nb_BF_1	143 ± 12	129 ± 12	281 ± 50
4.5Nb_GL_1	106 ± 16	213 ± 35	-
4.5Nb_GL_2	134 ± 15	182 ± 57	
4.5Nb_GL_3	154 ± 6	185 ± 37	
4.5Nb_BF_1	134 ± 2	89 ± 11	385 ± 58
4.5Nb_BF_2	169 ± 27	121 ± 27	446 ± 119
4.5Nb_BF_3	161 ± 26	98 ± 14	362 ± 66
6Nb_GL_1	132 ± 21	144 ± 28	-
6Nb_BF_1	118 ± 15	123 ± 42	

Conversely, the surface microstructures (SEM measured grain sizes are collected in Table 2) display marked differences depending on substrate, dopant concentration, and annealing conditions. For soda-lime glass, for which an optimal reaction temperature of 525 °C was identified, increasing the Nb content results in a progressive refinement of the surface morphology, manifested as progressively smaller vanadium oxide grain sizes, as measurements in Table 2 demonstrate. This trend may also be linked to the fact that higher dopant concentrations require shorter reaction times at a given temperature, thus limiting grain growth during oxidation. In contrast, coatings deposited on borosilicate substrates show a different evolution. A lower optimal reaction temperature (475 °C) was determined for this substrate, and increasing the Nb content from 3Nb to 4.5Nb under identical thermal conditions leads to significant changes in the surface morphology. Specifically, elongated surface features (which are distinctive of V_2_O_5_ ^37,38^ ) become increasingly prominent, emerging over a matrix of fine equiaxed grains (which averaged sizes are collected in Table 2, at the same column than that for the grain size of samples on soda-lime substrate). Moreover, unlike the case of soda-lime glass, higher dopant concentrations in borosilicate-supported films shift the optimal reaction window toward slightly longer reaction times. Although this does not induce major morphological changes, the matrix grains coarsen slightly while the elongated features (which equivalent diameter is shown, as “superficial feature size” at the last column in Table 2) become smaller and less pronounced. In this sense, even for samples 6Nb those features are not detected. A detailed grain and superficial feature size distribution for samples in Table 2 is present at the Supporting Information, Section II, in the form of histograms and plot fit to log-normal functions.

A different behavior is observed when examining the influence of reaction temperature. For borosilicate-supported samples, varying the annealing temperature within the studied range produces only minor microstructural changes. However, for soda-lime substrates, increasing the reaction temperature above 525 °C consistently promotes the formation of secondary surface structures, likely sodium vanadates, accompanied by a loss of surface uniformity. This behavior is consistent with enhanced Na diffusion at higher temperatures, a well-documented phenomenon in soda-lime glass substrates subjected to thermal treatments above 450–500 °C, where Na^+^ ions become mobile and diffuse toward the film surface. Once incorporated into the vanadium oxide layer, these species can react with V–O units, promoting the formation of Na–V–O secondary phases. This mechanism has been widely reported for oxide thin films grown on soda-lime glass, where Na diffusion significantly alters phase stability and composition.^18,39–41^ The presence of such Na-containing phases in the present work is further supported by the XRD and XPS results discussed below. Although direct quantification of Na diffusion by EDX remains challenging due to the low atomic number of sodium, complementary XPS depth-profiling analyses were performed to further elucidate its distribution and chemical state across the film thickness.

Overall, the oxidation time decreases with increasing temperature for both substrates. Nevertheless, this effect is more pronounced for soda-lime glass, resulting in a narrower processing window. Indeed, while higher Nb concentrations in borosilicate coatings generally require longer reaction times at a fixed temperature, the same adjustment cannot be applied to soda-lime glass. Extending the reaction time at elevated temperatures would promote Na diffusion and exacerbate vanadate formation, ultimately compromising the VO_2_(M) yield. This explains why a reaction temperature of 475 °C, requiring an optimal reaction time of 75 s for soda-lime glass, is not suitable for this substrate, in contrast to its effectiveness for borosilicate. Conversely, at 525 °C, a suitable balance between reaction time and temperature is achieved: the optimal reaction time decreases from 75 s (at 475 °C) to 10 s (at 525 °C) for the same nominal Nb content, limiting Na diffusion while enabling VO_2_(M) formation.

In summary, while borosilicate-supported coatings are primarily influenced by Nb concentration, soda-lime samples are additionally affected by the reaction temperature and time due to the role that these parameters play in Na diffusion. The choice of substrate therefore strongly conditions the resulting microstructure (grain size and homogeneity) as well as the final composition of the films. These factors are expected to significantly impact the optical and thermochromic performances of the coatings, as discussed in the following section.

Figure 3 shows the results of X-ray diffraction characterization regarding the evolution of the crystalline structure and phase composition for representative samples deposited on the two substrates, each annealed at its corresponding optimal oxidation temperature (525 °C, for soda-lime, Figure 3a; and 475 °C, for borosilicate, Figure 3b), as a function of increasing Nb concentration. In general, the X-ray diffractograms clearly indicate that VO_2_(M) (JCPDS card No. 82-0661; crystalline symmetry: *P*2_1_/*c*, international number: 14) is the predominant phase synthesized under optimal conditions for both substrates. Additional diffraction peaks associated with orthorhombic V_2_O_5_ (JCPDS card No. 89-0612; crystalline symmetry: *Pmmn*, international number: 59) are also detected, together with weaker reflections near 30° and 36°, which can be assigned to Na_1.1_V_3_O_7.9_ (JCPDS card No. 45-0498; crystalline symmetry: *P*2_1_/*m*; international number: 11) and V_6_O_13_ (JCPDS card No. 89-0100; crystalline symmetry: *C*2/*m*, international number: 12), respectively. In this regard, it seems that sodium vanadate signatures become more prominent at higher nominal Nb concentrations. They are detected even in coatings deposited on borosilicate substrates, which, although containing significantly less sodium than soda-lime glass, still retain trace Na levels sufficient to promote minor vanadate formation. In the presence of diffusing Na species, these Nb-induced modifications may facilitate the nucleation of Na–V–O secondary phases. Furthermore, the nonuniform spatial distribution of Nb, as revealed by STEM–EDX analyses (Figure 4, commented later on), likely generates regions with different local compositions and defect densities that can act as preferential sites for Na accumulation and subsequent vanadate formation. Therefore, Nb doping does not appear to directly enhance Na diffusion, but rather to modify the local thermodynamic and kinetic landscape governing phase stability in Na-containing environments. By contrast, V_6_O_13_ peaks tend to be more evident in samples with lower Nb content.

**Figure 3:**
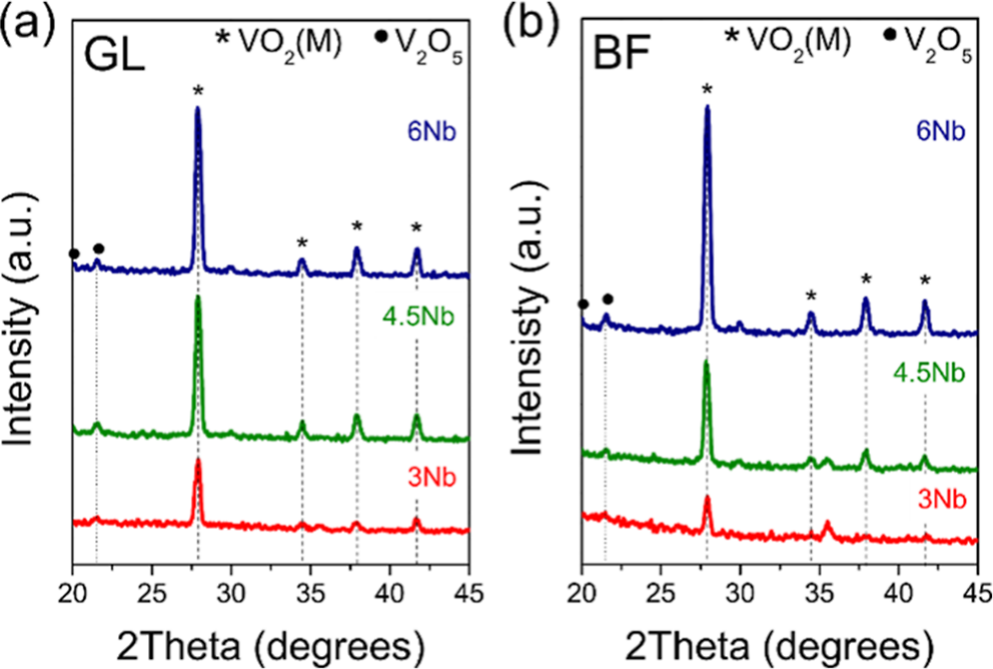
GIXRD patterns of Nb-doped VO_2_-based samples (3Nb, 4.5Nb, and 6Nb) fabricated on (a) soda-lime glass at 525 °C and (b) borosilicate glass at 475 °C.

**Figure 4:**
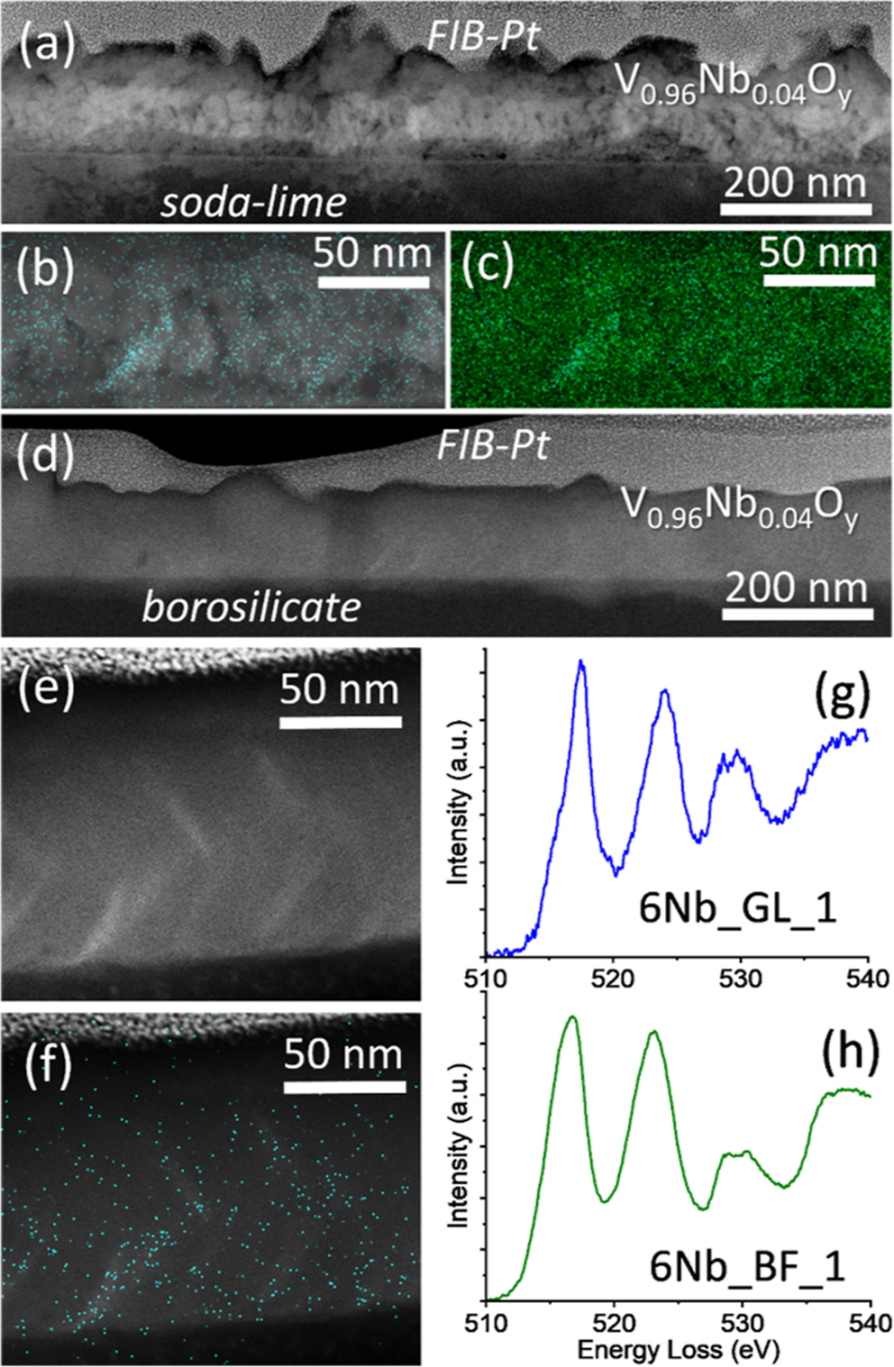
HAADF images of the Nb-doped vanadium oxide film in samples (a) 6Nb_GL_1 and (d) 6Nb_BF_1. EDX elemental maps (niobium in cyan; vanadium in green) for sample (b,c) 6Nb_GL_1. (e) HAADF image and (f) Nb-EDX elemental map for sample 6Nb_BF_1. Integrated EELS spectra for regions of the thin films for samples (g) 6Nb_GL_1 and (h) 6Nb_BF_1.

A particularly noteworthy trend is the progressive increase in the intensity relative to VO_2_(M), as well as an improvement in the overall crystallinity of the coatings, with increasing nominal Nb content for both substrates. While these intensities can be influenced by film thickness, it should be noted that all diffractograms were acquired under identical conditions and thickness variations remain limited (Table 2), suggesting that the observed crystallographic trends are representative of the films’ structural evolution. These observations are complemented by SEM and TEM analysis, which reveal consistent changes in grain morphology and phase distribution, in line with the structural trends inferred from GIXRD. This behavior may be related to a more stable development of the VO_2_(M) phase, possibly due to a more efficient and less constrained redistribution of Nb during annealing at higher dopant concentrations, where the dopant is more uniformly dispersed throughout the film. It is important to recall that the 6Nb series was deposited as a monolayer, suppressing the VO_*y*_/V_0.94_Nb_0.06_O_*y*_ interfaces present in the multilayer configurations that may promote local structural and compositional nonhomogeneities. Interestingly, this trend contrasts with what has been reported for W-doped VO_2_ films prepared following similar procedures,^32^ where higher W concentrations typically degrade crystallinity. This suggests that the observed behavior is not primarily dictated by the single-layer versus multilayer architecture but rather represents an intrinsic effect of Nb as a dopant.

Indeed, compositional STEM–EDX maps, such as the ones presented in Figure 4, further support the above interpretation by revealing distinct Nb distribution patterns compared with those observed for other dopants, such as W, in previous works.^32^ In this sense, Figure 4 collects representative compositional STEM results regarding samples 6Nb_GL_1 and 6Nb_BF_1. As can be observed through the comparison of HAADF images (Figure 4a,d), the use of a soda-lime substrate leads to the existence of a more granular texture, while the vanadium oxide fabricated on a borosilicate substrate presents wider areas where the coalescence of the grains has been produced. Also, EDX maps reveal regions where Nb atom placement preferentially follows the zigzag patterns. These can be observed in the overlapping of the HAADF image and EDX net map for Nb signal (in light blue) in Figure 4b,f. It is worth mentioning that, for sample 6Nb_BF_1, the zigzag pattern is visible in HAADF images at high enough magnification, since the higher averaged Z-number of V_*x*_Nb_1–*x*_O_*y*_ regions with lower values of “*x*” leads to higher intensities (brighter) in HAADF images. Therefore, Figure 4e (HAADF image that is overlapped with the Nb-EDX map in Figure 4f) is an example of how HAADF images reveal the pattern. Due to the more granular texture in sample 6Nb_GL_1, HAADF images such as the one overlapped in Figure 4b do not reveal that pattern so clearly, but the Nb EDX signal still does. Furthermore, also EDX maps obtained for samples with 4.5 and 6 Nb doping percentages reveal that the dopant is accumulated at higher atomic concentrations at regions closer to the substrate/film interface; this region thickness is typically a few tens-of-nanometers-thick. Such behavior can be observed at Table S3, at Supporting Information (Section II). In this table, the atomic Nb and V ratios, as percentages, are obtained from integrated EDX spectra for two regions (close to the substrate and close to the surface), while the last row of this table shows the same result for the whole film, serving as an average along the complete vanadium oxide layer.

On the other hand, vanadium EDX maps (Figure 4c is the overlap of V-signal, in green, and Nb-signal, light blue) reveal a more homogeneous and spread distribution of vanadium along the thin film. Regarding the oxidation state, it can be mentioned that integrated EELS spectra (Figure 4g,h), obtained from the thin films in 6Nb samples for both substrates, are in good agreement with a V^4+^ oxidation state (VO_2_), denoted by V-L_2,3_ peaks at 516–517 eV (L_3_) and 523–524 eV (L_2_) and 2 peaks in the O–K regions, corresponding to the 1s-t_2g_ (around 528 eV) and 1s-e_g_ (around 530 eV) transitions.^42,43^ Interestingly, and complementing the previous discussion on XRD, one could wonder if reasonably similar findings would arise in those samples where the layer architecture prior to the thermal treatment was a multilayered one instead of a single-layered one. After all, this might affect phenomena such as atom diffusion or MIT behavior. While this would require a dedicated work altogether using additional control samples, at least new STEM experiments were carried out in the other two key samples. Namely, we chose samples 4.5Nb_GL_2 and 4.5Nb_BF_1 to check whether the behavior of niobium does not change significantly. As advanced before, the results of this additional characterization are included in the Supporting Information of the present work and only summarized here (Table S3 and Figure S4). First, the heterogeneous distribution of the Nb atoms keeps taking place, since it is most abundant at roughly the bottom half of the V_*x*_Nb_1–*x*_O_*y*_ structure. Second, the V^4+^ oxidation state is found successfully again. Therefore, one could at least expect in principle that the dopant behaves somewhat similarly regardless of the initial layer architecture.

HAADF images such as the ones in Figure 4a,d and also BFTEM micrographs (see Figure 5) allowed the thickness measurement of samples 6Nb_GL/BF_1 oxide film as reported in Table 2. The granular microstructure in sample 6Nb_GL_1 may be observed thanks to the intensity contrast in the HAADF images. It is clear that a thicker granular formation is present at the lower part of the film (thus, the higher intensity in Z-contrast STEM images, which is also proportional to the material thickness along the electron beam path). The equivalent diameters of these grains range between 15 and 35 nm. The upper region of this granular structure that can be observed in Figure 4a is not as compact as the lower part of the film, which is in good agreement with the superficial features appearing in the SEM image in Figure 2.

**Figure 5:**
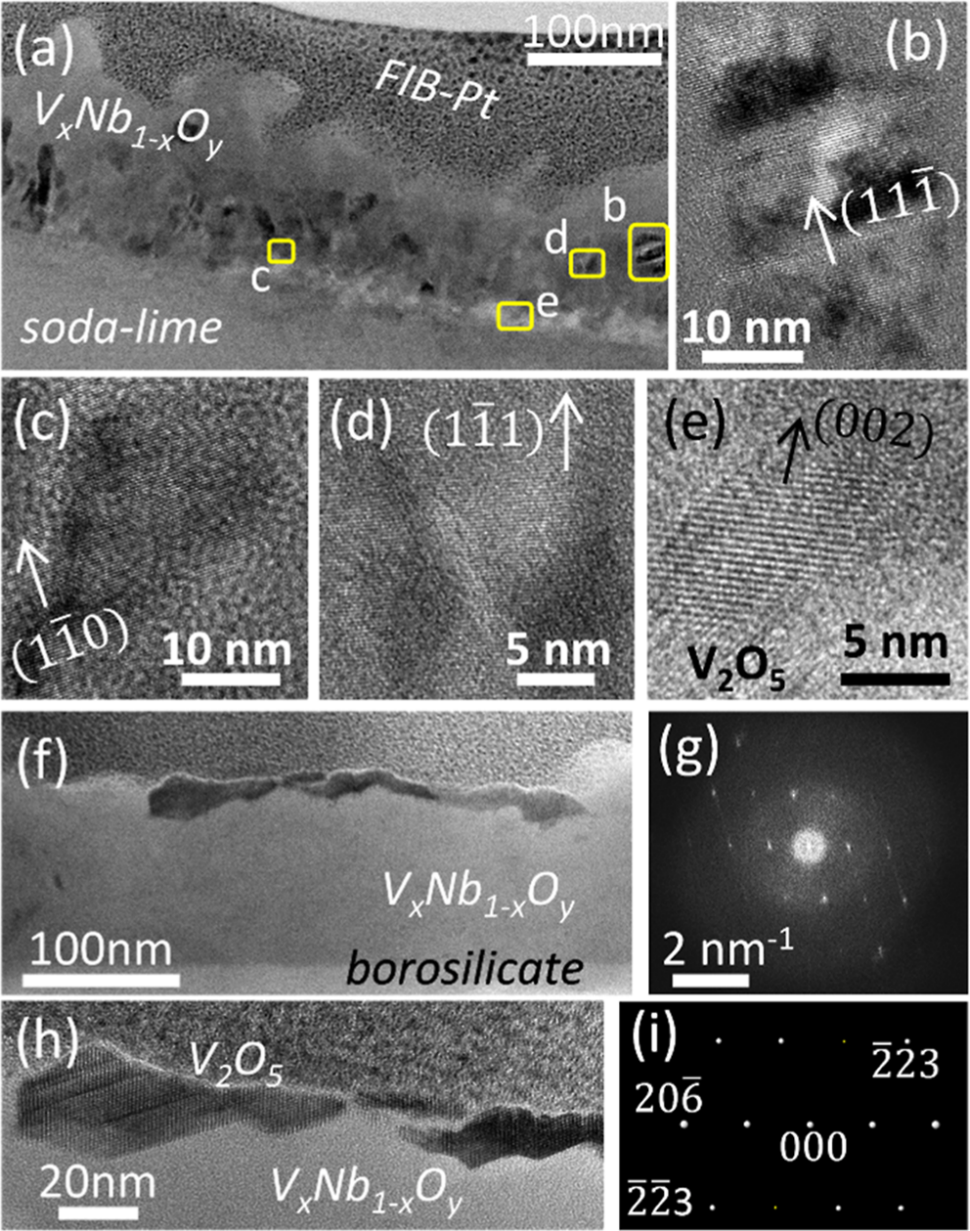
BFTEM micrographs for the thin film in samples (a) 6Nb_GL_1 and (f) 6Nb_BF_1. HRTEM images from nano- and micrometric regions in the BFTEM micrographs, showing atomic structures of (b–d) VO_2_ and (e,h) V_2_O_5_. (g) Experimental and (i) simulated fast Fourier transform, obtained from the HRTEM image in (h).

Figure 5a,f shows BFTEM images acquired over micrometric regions of samples 6Nb_GL_1 and 6Nb_BF_1, respectively, from which HRTEM analyses were performed. The intensity contrast observed in the BFTEM micrographs reveals the presence of domains with different crystallographic orientations. In particular, Figure 5a demonstrates that the granular structure observed in Figure 4a is polycrystalline mainly in the thicker lower region of the film, whereas the upper region and the film deposited on borosilicate glass exhibit a more uniform crystallographic character. This is in good agreement with the affirmations regarding the structural coalescence for the vanadium oxide deduced from SEM (Figure 2) and HAADF-STEM (Figure 4d) analyses.

Finally, electron microscopy also confirmed the presence of vanadium oxide regions with different oxidation states through structural measurements, namely, HRTEM results. Figure 5 shows examples of HRTEM images for nanometric and micrometric single crystals of the vanadium oxides detected by XRD (Figure 3). In this sense, in Figure 5a, 4 yellow-framed areas indicate nanometric regions from which HRTEM images have been obtained, associated with Figure 5b–e. The atomic plane spacing measured in such figures is in good agreement with the theoretical atomic distances for VO_2_(M) atomic planes (111̅), (11̅0), and (11̅1) and for the orthorhombic V_2_O_5_ (001) plane. Figure 5h also displays a crystalline micrometric area from the oxide film in 6Nb_BF_1 sample, visible at lower magnification in Figure 5f. The monocrystalline nature of this large region is also demonstrated by the existence of a crystalline defect: stacking fault. The corresponding FFT (Figure 5g) confirms that this region can be unambiguously assigned to V_2_O_5_, as the experimental pattern matches only the simulated FFT of the [−6 3 2] zone axis of this phase (Figure 5i).

To complement the structural and compositional analyses obtained by SEM, STEM–EDX, and GIXRD, a comprehensive XPS study was performed on representative 6Nb coatings deposited on soda-lime and borosilicate substrates. The objective was to elucidate (i) the oxidation states of V and Nb, (ii) the presence and depth distribution of Nb and Na from the film surface down to the film/substrate interface, and (iii) the overall composition of the films on each substrate. The combined results are summarized in Figure 6. For both samples, the survey spectra and the atomic/relative percentages (left/right axes) of every element are shown in Figure 6a,d, respectively. The elements detected in all cases are V, O, Na, and C. Notably, Na is clearly present at the surface of both films, although its relative intensity differs depending on the substrate, whereas Nb is not detected at the surface in either sample. This observation is fully consistent with the STEM–EDX results, which revealed that Nb preferentially accumulates in the lower region of the film rather than near the surface. The relative ratios extracted from Figure 6d further highlight these substrate-dependent differences: for the soda-lime sample, the Na/V ratio reaches ∼0.20 at the surface, confirming the strong Na enrichment caused by diffusion from the substrate, whereas the borosilicate sample exhibits a much lower Na/V ratio of ∼0.045, consistent with the negligible Na content of this substrate. The Nb/V ratio is not observable at the surface for either sample because Nb remains below the detection limit in the as-fabricated state; however, once Nb becomes detectable during etching, the Nb/V ratio reaches ∼0.14 for the soda-lime sample and ∼0.08 for the borosilicate sample.

**Figure 6:**
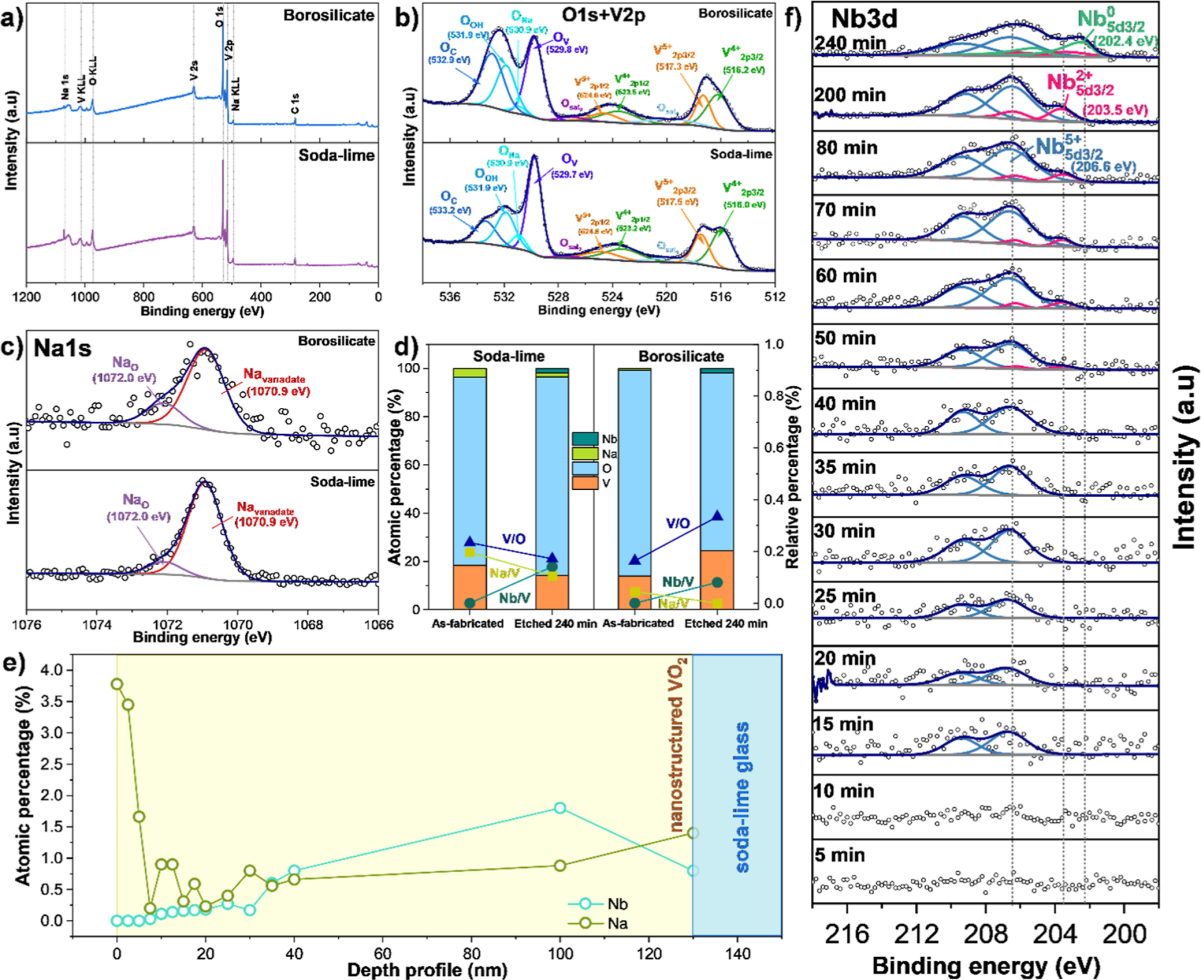
(a) XPS survey spectra of 6Nb-VO_2_ coatings deposited on soda-lime and borosilicate glass substrates. (b) High-resolution O 1s and V 2p core-level spectra. (c) High-resolution Na 1s core-level spectra. (d) Atomic concentrations (left axis) and relative atomic percentages (right axis) obtained from the XPS analysis of as-fabricated and etched samples on both substrates. (e) XPS depth profiles of Nb and Na atomic concentrations as a function of film thickness for the soda-lime glass sample. (f) Evolution of high-resolution Nb 3d core-level spectra during depth profiling.

The high-resolution V 2p + O 1s spectra (Figure 6b) show that, at the surface, vanadium is present as both V4^+^ and V5^+^, with V 2p_3/2_ components located at approximately 516.2 and 517.3 eV, respectively, separated by 7.33 eV from their corresponding V 2p_1/2_ peaks. The presence of V^5+^ is attributed to the formation of overoxidized vanadium species (V_2_O_5_, V_6_O_13_) as well as sodium vanadates generated by Na^+^ ions diffusing from the substrate toward the top of the film. Since it is not possible to deconvolute which fraction of the V^5+^ signal corresponds to vanadates and which to vanadium oxides solely from the V 2p region, a high-resolution deconvolution of the Na 1s peak was performed for both substrates (Figure 6c). This analysis reveals two components: one at 1070.9 eV, assigned to Na–V bonding associated with sodium vanadate formation, and a second, much smaller component at 1072.0 eV, attributed to Na–O bonding in surface oxides/hydroxides.^44,45^ Quantification of these contributions indicates that approximately 55% and 15% of the V^5+^ present at the surface correspond to sodium vanadates for the soda-lime and borosilicate substrates, respectively, while the remaining fraction arises from overoxidized vanadium oxides (V_2_O_5_, V_6_O_13_). In the same spectral region, the O 1s envelope provides additional insight into the chemical environment of oxygen. Both samples exhibit a dominant peak at ∼529.7 eV, assigned to lattice oxygen in V–O bonds (O_V_). A smaller component at 530.9 eV, attributed to Na–O bonding (O_Na_), is also present and reinforces the formation of Na-containing oxides/hydroxides discussed above. Two additional secondary components appear at ∼531.9 eV and ∼532.9 eV, corresponding to surface hydroxyl groups (O_OH_) and oxygen bound to adsorbed organic species or water (O_C_), respectively. The latter is noticeably more intense for the borosilicate sample, likely due to its lower Na content and the consequent reduced formation of Na-vanadate species, which leaves a larger fraction of the surface available for adsorption of adventitious carbon and moisture.

To confirm how both Nb and Na are distributed throughout the film, a detailed depth-profiling analysis was performed on the 6Nb coating deposited on soda-lime glass. This was achieved by progressively etching the sample using an Ar^+^ ion gun at successive times, allowing the chemical composition to be monitored from the film surface down to the film/substrate interface. The etching rate of the ion gun was calibrated by measuring the time required to completely sputter a film of known thickness until the appearance of Si peaks from the soda-lime substrate in the XPS spectrum. Under these conditions, the complete removal of the film occurred after 240 min, corresponding to an etching rate of approximately 0.5 nm min^–1^. Figure 6e displays the resulting depth profile. At the top of the film (0 nm), the surface is clearly enriched in Na^+^, confirming that sodium diffuses from the soda-lime substrate toward the outermost region of the coating during thermal treatment. As etching progresses, the Na signal decreases sharply within the first 7–10 nm, precisely when the Nb signal begins to emerge. In the intermediate region between approximately 10 and 100 nm, the Na concentration remains nearly constant, whereas the Nb signal increases steadily, reaching a maximum atomic percentage of about 2%. In the final stage of the profile, the Nb signal decreases while the Na signal rises again, indicating that the VO_2_ layer has been almost completely removed and that the analysis is approaching the soda-lime glass surface. The survey spectrum obtained after 240 min of etching, in which the Si substrate signal becomes dominant, is shown in Supporting Information, Section II (Figure S5). The XPS depth profile complements the distribution previously measured by STEM–EDX: Na shows a strong interfacial enrichment and a residual presence toward the surface, whereas Nb is absent at the top of the film and increases progressively with depth until reaching its maximum near the interface. The agreement between both techniques confirms the existence of a vertical chemical gradient driven by Na diffusion from the soda-lime substrate and by the preferential segregation of Nb toward the bottom of the VO_2_ layer. This Na redistribution is consistent with the well-known mobility of Na^+^ in soda-lime glass at temperatures above 450–500 °C, where it can diffuse toward the film and react with oxidized vanadium species. Moreover, Na incorporation is thermodynamically more favorable in V_2_O_5_-rich environments, and the presence of grain boundaries and microvoids in the VO_2_ layer provides effective diffusion pathways for Na to reach these regions.^46,47^ For the borosilicate sample, a similar depth-profiling experiment was performed. The Nb atomic percentage reached the same maximum value (∼2 at.), indicating that the incorporation efficiency of Nb into the VO_2_ lattice is comparable on both substrates. It should be noted that the Nb atomic percentages obtained from XPS are lower than those derived from STEM–EDX (∼4 at. for the same sample). This difference is attributed to the distinct sampling volumes and quantification sensitivities of both techniques, as well as to the spatially nonuniform distribution of Nb within the film, rather than to a contradiction between the two measurements.

Figure 6f showcases the evolution of the Nb 3d spectral region during the XPS depth profile of the 6Nb coating. From the first detectable appearance of Nb within the film, the Nb 3d signal corresponds exclusively to Nb^5+^, with the characteristic Nb 3d_5/2_ component at ∼206.6 eV. This confirms that Nb is incorporated in the desired Nb^5+^ oxidation state required for substitutional doping in the VO_2_ lattice. In this configuration, Nb^5+^ replaces V^4+^, acting as an electron donor and contributing to the reduction of the MIT temperature.^48^ More broadly, the substitution of Nb^5+^ for V^4+^ introduces a local charge imbalance and lattice distortions within the VO_2_ structure, which may contribute to defect formation and influence the relative stability of the monoclinic phase, as previously discussed for VO_2_ and related oxide systems.^49–51^

As etching progresses, an additional lower-binding-energy component assigned to Nb^2+^ appears after ∼50 min of Ar^+^ etching, while Nb^5+^ remains the dominant species. This partial reduction is attributed to sputter-induced oxygen loss during ion bombardment. At the final stages of the depth profile, a Nb^0^ contribution also becomes detectable as a consequence of further sputter-induced reduction under prolonged etching conditions. Thus, the progressive evolution from Nb^5+^ to Nb^2+^ and finally Nb^0^ directly reflects the chemical reduction induced by the XPS etching process.

### 3.3 Optical-Thermochromic Performance and Switching Kinetics

Following the comprehensive microstructural and compositional characterization, the functional performance of the V_*x*_Nb_1–*x*_O_2_-based films deposited on the two distinct substrates was rigorously evaluated for smart-glazing applications. UV–vis–NIR transmittance spectra (300–2500 nm) were recorded for the optimal subset of samples in both the semiconductor (M1 phase, 10 °C) and metallic (R phase, 90 °C) states, as shown in Figure 7. The corresponding photometric (*T*_lum_) and radiometric (Δ*T*_sol_) key parameters derived from these measurements are summarized in Table 3.

**Figure 7:**
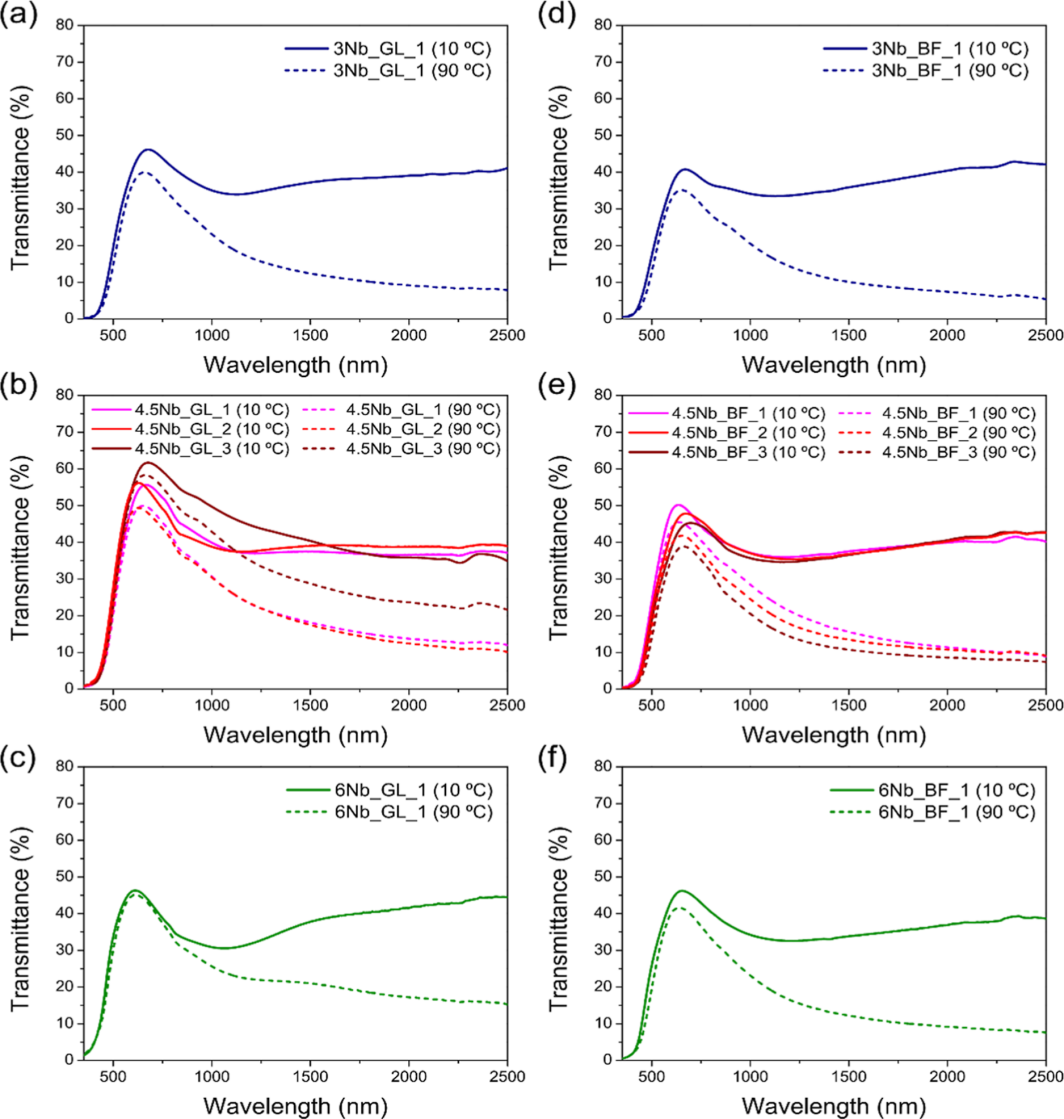
UV–vis–NIR transmittance spectra of V_*x*_Nb_1–*x*_O_2_-based coatings recorded at 10 °C (M1 phase, solid lines) and 90 °C (R phase, dashed lines). Spectra are grouped by substrate type: (a–c) soda-lime glass and (d–f) borosilicate glass. Panels are organized by increasing Nb doping: (a,d) 3Nb, (b,e) 4.5Nb, and (c,f) 6Nb. In the 4.5Nb samples (b,e), the colors indicate the postdeposition annealing temperature: 475 °C (magenta), 525 °C (red), and 550 °C (wine).

**Table 3:** Radiometric and Photometric Parameters Upon Heating for the Optimal Sample Selection in This Study^a^

Sample	*T*_lum_ (%)	Δ*T*_lum_ (%)	Δ*T*_sol_ (%)
3Nb_GL_1	30.8	4.2	9.4
3Nb_BF_1	29.1	3.5	9.7
4.5Nb_GL_1	38.9	3.4	7.5
4.5Nb_GL_2	40.4	4.1	7.9
4.5Nb_GL_3	43.3	1.4	4.7
4.5Nb_BF_1	37.1	4.1	7.6
4.5Nb_BF_2	32.6	3.2	9.2
4.5Nb_BF_3	29.1	3.6	10.4
6Nb_GL_1	40.8	1.8	4.9
6Nb_BF_1	34.7	4.0	8.6

a The accuracy of these values is ±0.1%.

*T*_lum_ was calculated as (eq 1)­

1 $$T_{lum}=\frac{\int_{380}^{780}T\left(\lambda \right)\cdot \phi_{lum}\left(\lambda \right)d\lambda }{\int_{380}^{780}\phi_{lum}\left(\lambda \right)d\lambda }$$

where *T*(λ) is the spectral transmittance and Φ_lum_(λ) is the photopic response of the human eye (CIE 1931 standard observer).

Similarly, the solar transmittance (*T*_sol_) was obtained as (eq 2)­

2 $$T_{sol}=\frac{\int_{300}^{2500}T\left(\lambda \right)\cdot \phi_{sol}\left(\lambda \right)d\lambda }{\int_{300}^{2500}\phi_{sol}\left(\lambda \right)d\lambda }$$

where Φ_sol_(λ) corresponds to the AM1.5 solar irradiance spectrum. Luminous transmittance variations (Δ*T*_lum_) and solar modulation efficiency (Δ*T*_sol_) were obtained from the difference in *T*_lum_ and *T*_sol_ between the semiconductor (10 °C) and metallic (90 °C) states.

A first comparison of the spectral signatures reveals clear differences in the optical behavior of coatings fabricated on soda-lime versus borosilicate glass. For soda-lime substrates (Figure 7a–d), samples annealed at the optimal reaction temperature of 525 °C exhibit a progressive reduction in the NIR modulation (Δ*T* around ∼1000 nm) as the nominal Nb content increases. This trend directly translates into a systematic decrease in Δ*T*_sol_ from 9.4% (3Nb) to 4.9% (6Nb), as listed in Table 3. Furthermore, for a fixed dopant concentration (Figure 7b), annealing at temperatures exceeding 525 °C results in a noticeable increase in visible transmittance but a sharp deterioration of the VO_2_ yield in favor of secondary phases. Consequently, the thermochromic contrast decreases across the entire vis–NIR range, reducing Δ*T*_sol_ from 7.9% to 4.7% when increasing the annealing temperature from 525 to 550 °C (compare 4.5Nb_GL_2 and 4.5Nb_GL_3 in Table 3).

These observations are fully consistent with the microstructural and compositional analyses discussed previously, suggesting that higher-temperature annealing exceeds the stability window of the VO_2_ stoichiometry on soda-lime glass. This promotes not only Na diffusion from the soda-lime substrate and the likely formation of sodium vanadates but also the progressive formation of more oxidized vanadium species (i.e., V_2_O_5_ and related VO_2+*x*_ phases). This compositional shift enhances luminous transmittance through the formation of higher-valence oxides that act as a more transparent but nonthermochromic matrix, thereby leading to a concomitant deterioration of Δ*T*_sol_ by reducing the volume fraction of the active VO_2_(M) phase. Accordingly, these results underscore that, under the present processing conditions, the formation of secondary VO_*x*_ phases cannot be completely avoided but can be effectively minimized within a narrow oxidation window, which ultimately governs the balance between luminous transmittance and thermochromic performance. Finally, it should also be noted that while variations in film thickness may contribute to the observed optical response, thickness in this study is intrinsically linked to the oxidation degree, microstructure, and phase composition. Consequently, the optical behavior is primarily governed by the relative fraction of VO_2_(M) and secondary phases rather than by thickness alone.

In contrast, films deposited on borosilicate substrates exhibit a far more robust and reproducible functional response. For samples annealed at the optimal temperature of 475 °C, these spectra remain practically similar regardless of Nb content (Figure 7d–f), suggesting that the thermochromic performance is mostly independent of dopant concentration. When the annealing temperature is increased (Figure 7e), a systematic red-shift of the visible-range transmittance maximum is observed. This leads to slight decreases in *T*_lum_ but enhances Δ*T*_sol_, in line with the classical trade-off between these two parameters in VO_2_-based coatings (see 4.5Nb_BF series in Table 3).

Overall, in agreement with the conclusions previously drawn, the combined evaluation of *T*_lum_ and Δ*T*_sol_ highlights that Nb-doped VO_2_ coatings deposited on borosilicate glass offer greater processing robustness than those fabricated on soda-lime glass. In soda-lime substrates, the interaction between the dopant content and annealing conditions leads to a progressive narrowing of the optimal reaction window; annealing above 525 °C or increasing the Nb content both exacerbate the formation of secondary phases and reduce the solar-modulation efficiency. This behavior contrasts sharply with borosilicate substrates, for which the optical performance remains largely insensitive to Nb concentration and only moderately affected by annealing conditions.

This trend is further supported by the extended data set included in the Supporting Information, Section I. For the 6Nb composition, only one additional sample deposited on soda-lime glass exhibited Δ*T*_sol_ > 4%, whereas five more samples on borosilicate glass met this criterion, despite an identical number of deposition and annealing trials. Finally, and unlike what has been previously reported for W-doped VO_2_,^32^ the reduction (more moderate for films on borosilicate glass) in Δ*T*_sol_ observed at higher Nb concentrations is not accompanied by a loss in *T*_lum_ for either substrate; in some cases, a slight improvement in visible transmittance is even detected.

To assess the reversible optical MIT hysteresis of the Nb-doped VO_2_ coatings and extract the corresponding kinetic parameters, temperature-dependent transmittance measurements were carried out at a fixed wavelength of 2500 nm over consecutive heating–cooling cycles between 15 and 90 °C. Representative results for films deposited on soda-lime and borosilicate substrates are shown in Figures 8 and 9, respectively. The first three images in these figures (Figures 8a–c and 9a–c) depict the thermal evolution of optimal transmittance using soda-lime glass and borosilicate glass substrates, respectively, whereas Figures 8d–f and 9d–f compile their associated derivatives. The characteristic hysteresis parameters derived from these measurements are summarized in Table 4. Because of the complex MIT response observed in several samples, it is important to clarify the procedure used to extract the transition temperatures. Derivative curves were obtained from the transmittance–temperature plots and fitted with Gaussian functions. All discernible peaks, both primary and secondary, were considered, and a weighted average based on the integrated area of each was used to determine a representative *T*_c_. Unless otherwise specified, any reference to *T*_c_ in the following discussion refers to the critical temperature during heating.

**Figure 8:**
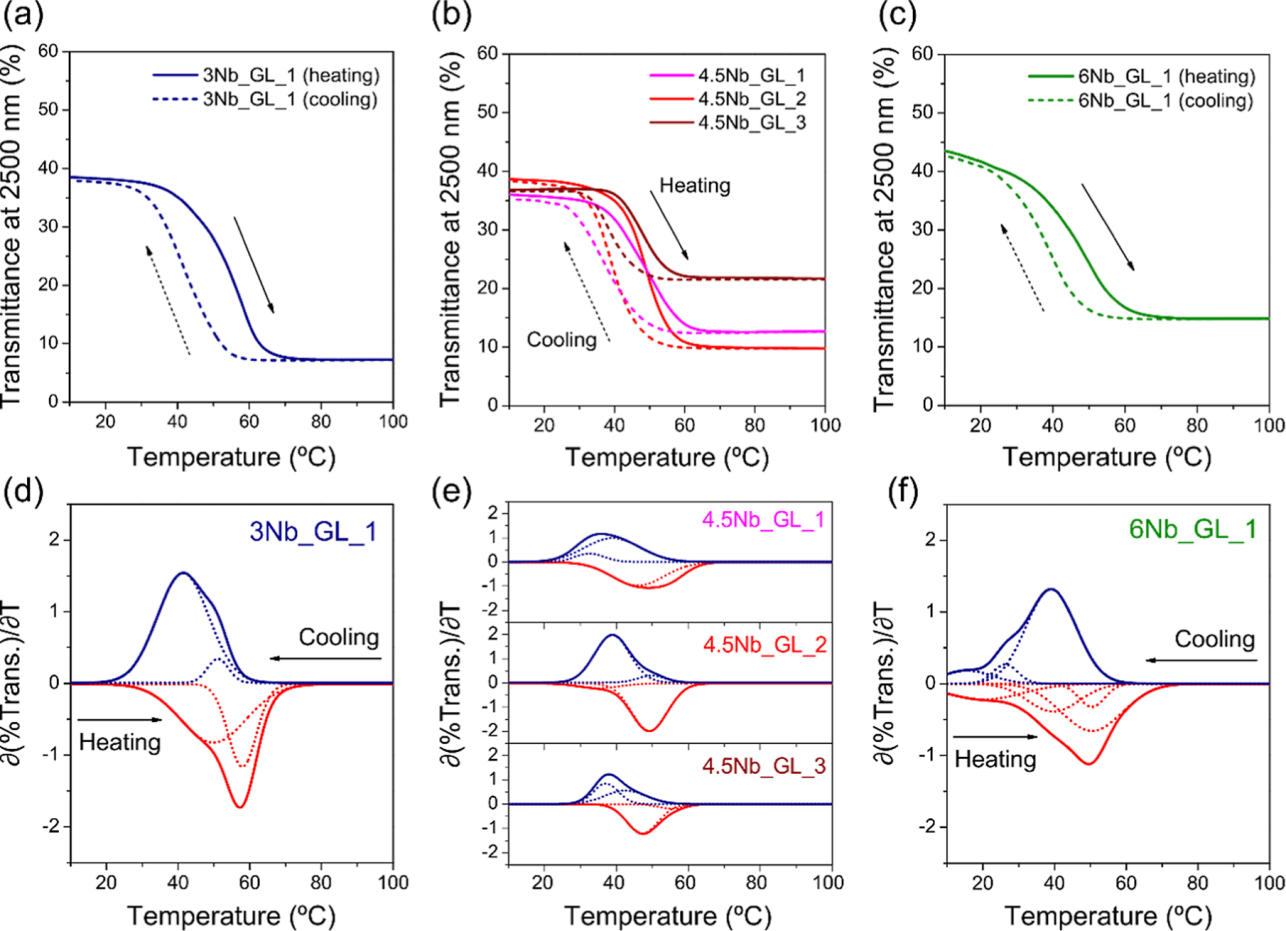
(a–c) Thermal evolution of the optical transmittance at 2500 nm recorded during consecutive heating (solid lines) and cooling (dashed lines) cycles together with (d–f) their associated derivatives (red and blue lines, respectively) for the optimal sample selection on soda-lime glass. For a better overview, the cooling derivatives are represented in absolute values.

**Figure 9:**
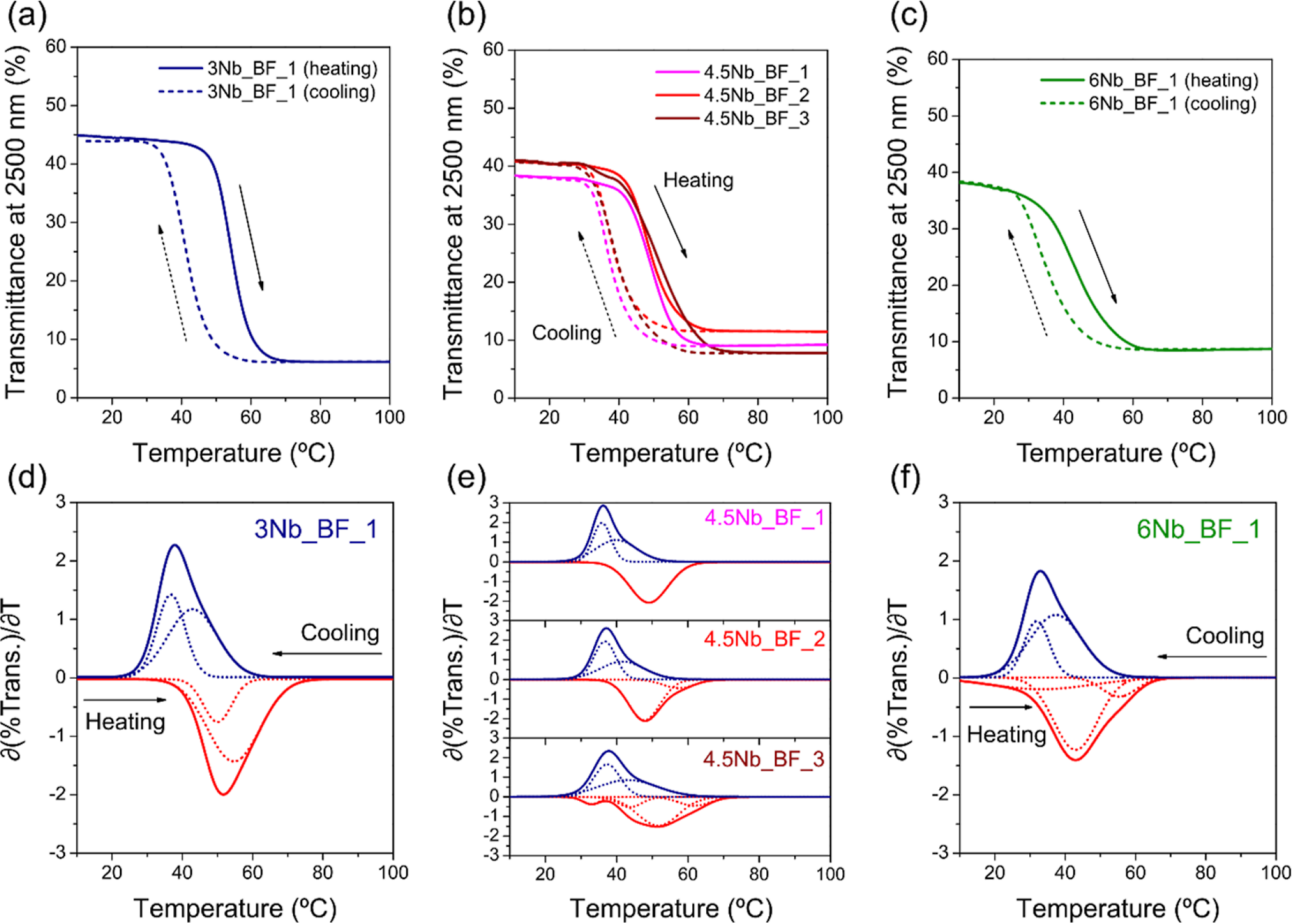
(a–c) Thermal evolution of the optical transmittance at 2500 nm recorded during consecutive heating (solid lines) and cooling (dashed lines) cycles together with (d–f) their associated derivatives (red and blue lines, respectively) for the optimal sample selection on borosilicate glass. For a better overview, the cooling derivatives are represented in absolute values.

**Table 4:** Main Features of the Thermochromic Hysteresis Loops Changes Upon Heating–Cooling Cycles for the Optimal Sample Selection in This Study^a^

Sample	*T*_c_ (°C)	*W*_H_ (°C)	*T*_c,avg_ (°C)	Δ*T*_rel_ (%)
3Nb_GL_1	53.0	10.5	48.0	81.1
3Nb_BF_1	53.5	14.0	46.5	86.2
4.5Nb_GL_1	48.0	10.0	43.0	64.7
4.5Nb_GL_2	48.0	8.0	44.0	74.7
4.5Nb_GL_3	48.0	8.0	44.0	41.5
4.5Nb_BF_1	49.0	11.0	43.5	76.0
4.5Nb_BF_2	49.5	10.5	44.0	71.9
4.5Nb_BF_3	51.0	11.0	45.5	81.2
6Nb_GL_1	42.0	6.5	39.0	65.7
6Nb_BF_1	42.0	6.5	39.0	77.2

a *T*_c_ denotes the temperatures of the MIT transition on heating (°C); *W*_H_ is the hysteresis loop width given by the differences of the MIT temperatures on heating and cooling cycles (°C); *T*_c,avg_ is the average MIT temperature on heating and cooling cycles (°C); and Δ*T*_rel_ is the relative decrease of transmittance upon the transition at 2500 nm. The accuracy of temperature and transmittance values are ±0.5 °C and ±0.1%, respectively.

For coatings deposited on soda-lime substrates, the heating–cooling curves in Figure 8 reveal a clear and monotonic decrease in *T*_c_ with increasing Nb content, confirming the expected dopant-induced reduction of the MIT temperature. As shown in Table 4, *T*_c_ decreases from 53 °C (3Nb) to 42 °C (6Nb). However, this beneficial shift is accompanied by the emergence of multistep transitions, manifested as multiple peaks in the derivative curves. This behavior is consistent with the preferential accumulation of Nb within specific regions of the film thickness, as previously evidenced by the STEM–EDX elemental maps, yielding domains with different local dopant levels. Importantly, this multistep character cannot be attributed to the multilayer configuration itself since the 6Nb film, deposited as a monolayer, exhibits the most pronounced peak multiplicity. Nevertheless, it should be acknowledged that the use of different deposition configurations (multilayer for 3Nb/4.5Nb and single layer for 6Nb) may introduce additional effects on diffusion and crystallization. While a complete decoupling of these factors is beyond the scope of this work, the consistency of the observed trends across all samples suggests that Nb incorporation remains the dominant factor governing the structural and functional responses.

At a fixed nominal Nb content, the influence of the annealing temperature is also apparent. For the 4.5Nb series, the derivative curve corresponding to the sample annealed at 525 °C displays sharper and more localized peaks, highlighting once again that this is the optimal reaction temperature for soda-lime substrates (Figure 8e). This observation is further supported by the evolution of Δ*T*_rel_, which provides a qualitative indication of the fraction of VO_2_(M) effectively participating in the transition. Moreover, the hysteresis width (*W*_H_) systematically narrows with increasing Nb concentration, in agreement with typical trend reported in the literature.^27,35,52^

The general trends observed for soda-lime substrates are retained in the borosilicate-supported films, yet their manifestation is significantly moderated. As shown in Figure 9 and Table 4, both *T*_c_ and *W*_H_ decrease with increasing Nb content, following nearly identical numerical values to those obtained for soda-lime glass (from 53.5 to 42 °C for *T*_c_ and from 14.0 to 6.5 °C for *W*_H_). Likewise, the derivative curves broaden progressively as the dopant content increases, reflecting the appearance of multistage transitions linked to imperfect dopant homogeneity.

However, these effects are far less pronounced than in the soda-lime samples. For instance, the 6Nb_BF derivative curve (Figure 9f) exhibits considerably fewer secondary features than its soda-lime counterpart (Figure 8f), i.e., a noticeably sharper and more localized MIT, suggesting that Nb diffuses and redistributes more effectively during annealing on borosilicate glass. This behavior is consistent with the more limited Na diffusion in this substrate, which enables the use of higher reaction temperatures and longer oxidation times without promoting substantial phase degradation. A notable exception is the sample annealed at 550 °C (4.5Nb_BF_3), which exhibits a particularly broad and irregular derivative curve (Figure 9b,e). This can be due to the fact that, although VO_2_(M) is formed at this temperature, the annealing time is insufficient to ensure homogeneous Nb diffusion, leading to noticeable variations in the local doping level.

These findings, together with the structural and optical analyses, indicate that borosilicate glass inherently provides a more favorable environment for stabilizing high-quality VO_2_-based thermochromic coatings. Nevertheless, this enhanced performance should not be interpreted as an argument for using borosilicate substrates in practical smart-window applications. Their current higher cost and lower industrial relevance make soda-lime glass the only feasible option at large scale. The present results therefore highlight two important considerations. First, even substrates with broadly similar compositions can lead to substantial differences in microstructure, dopant distribution, and functional behavior, underscoring the need to account for substrate effects when evaluating MIT characteristics. Second, improving VO_2_-based coatings for real-life glazing requires strategies that specifically address the limitations of soda-lime glass, most notably, Na diffusion. In this context, barrier interlayers, such as compact SiO_2_ films (50–100 nm) deposited through scalable methods, have proven effective in suppressing Na migration and thereby restoring a broader processing window.^53^ Furthermore, these observations call into question the relevance of studies performed on idealized substrates such as sapphire or quartz for assessing the technological viability of VO_2_ (either doped or undoped) in smart-glazing platforms. As demonstrated here, fabrication optimized on such substrates may not translate to soda-lime glass, potentially leading to overly optimistic conclusions about their applicability. In any case, when evaluated in terms of the overall balance between *T*_lum_, Δ*T*_sol_, and *T*_c_, the performance metrics achieved in this study generally match or surpass those previously reported in the literature using Nb as a dopant,^17,24,35^ even in works employing less realistic substrates for smart-window applications.^54^ This represents a meaningful step forward in the development of VO_2_-based thermochromic technologies.

When comparing the results obtained for Nb-doped VO_2_ coatings deposited on conventional soda-lime glass with those previously reported for W-doped VO_2_ films fabricated using a similar deposition methodology and the same type of substrate,^32^ several relevant differences emerge. Table 5 summarizes the main differences between Nb- and W-doped VO_2_ coatings under comparable processing conditions. Although Nb induces a significantly lower reduction in the transition temperature than W, approximately 11 °C per Nb atom percentage unit increase (based on the V_*x*_A_1–*x*_O_2_ formulation and referenced to undoped films processed under identical conditions), compared with about 20 °C per W atom percentage unit increase, the use of Nb does not lead to a deterioration of luminous transmittance as the dopant concentration increases, but rather the opposite (i.e., it generally increases). A similar trend is observed for the solar modulation efficiency. While Δ*T*_sol_ decreases with increasing dopant content for both elements, this degradation is noticeably less severe for Nb. In practice, nearly twice the dopant concentration is required in the case of Nb to reach a reduction in Δ*T*_sol_ comparable to that induced by W (6Nb versus 3W to reach Δ*T*_sol_ ∼ 5%). It is also worth noting that, for analogous fabrication conditions (i.e., multilayers), W tends to diffuse more homogeneously than Nb during thermal treatments. As a result, Nb-doped films often exhibit more step-like or multistage hysteresis behavior, which can be regarded as an intrinsic characteristic of this dopant rather than a consequence of the deposition strategy.

**Table 5:** Side-by-Side Comparison between Nb- and W-Doped VO_2_ Coatings Deposited on Soda-Lime Glass (This Work vs Santos et al.^32^)

Parameter	Nb-doped VO_2_ (this work)	W-doped VO_2_ (Santos et al.^32^)
*T*_c_ reduction (°C/doping percentage unit)	∼11	∼20
*T*_lum_ trend vs doping	increases	decreases
Δ*T*_sol_ trend vs doping	moderate decrease	strong decrease
Nominal doping for Δ*T*_sol_ ∼ 5%	∼6 at. % Nb	∼3 at. % W
Hysteresis behavior	multistep/broader	more uniform
Dopant diffusion	less homogeneous	more homogeneous
Structural perturbation	moderate	strong
Practical implication	better optical balance	more efficient *T*_c_ tuning

From a technological perspective, Table 5 highlights a clear trade-off between both dopants: W doping provides a more efficient reduction of *T*_c_, whereas Nb offers a more balanced combination of luminous transmittance and solar modulation efficiency, which is advantageous for smart-glazing applications. These trends are consistent with previous reports on Nb-doped VO_2_, where Nb^5+^ acts as an electron donor that modifies the electronic structure while preserving favorable optical properties such as luminous transmittance and solar modulation efficiency and maintaining a stable thermochromic response.^25,27,55^ Compared to W doping, which induces a stronger lattice distortion due to its higher valence state (W^6+^), Nb results in a more moderate perturbation of the VO_2_ structure, thereby limiting the degradation of Δ*T*_sol_ at increasing dopant concentrations.

However, both niobium and tungsten are classified as critical raw materials in major frameworks such as the EU^56^ and USGS^57^ lists, although their supply chains differ. This means that the choice between them is mainly governed by performance-stability trade-offs rather than availability. Although systematic long-term aging studies remain limited, Nb doping has been associated in the literature with a milder structural perturbation and improved stability of the thermochromic response under cycling conditions compared with heavily W-doped VO_2_ systems.^7,58^

Finally, Figure 10 presents the temperature-dependent UV–vis–NIR transmittance spectra of the 6Nb coatings deposited on both substrates (Figure 10a–c,d–f for soda-lime glass and borosilicate glass-based samples, respectively), collected during sequential heating and cooling cycles, together with the evolution of Δ*T*_sol_ as a function of sample temperature. The latter is obtained by calculating Δ*T*_sol_ at each temperature, always using the spectrum recorded at 10 °C as a reference. This way of illustrating the results enables a final and broader reflection on the current state-of-the-art of VO_2_-based smart-window technologies.

**Figure 10:**
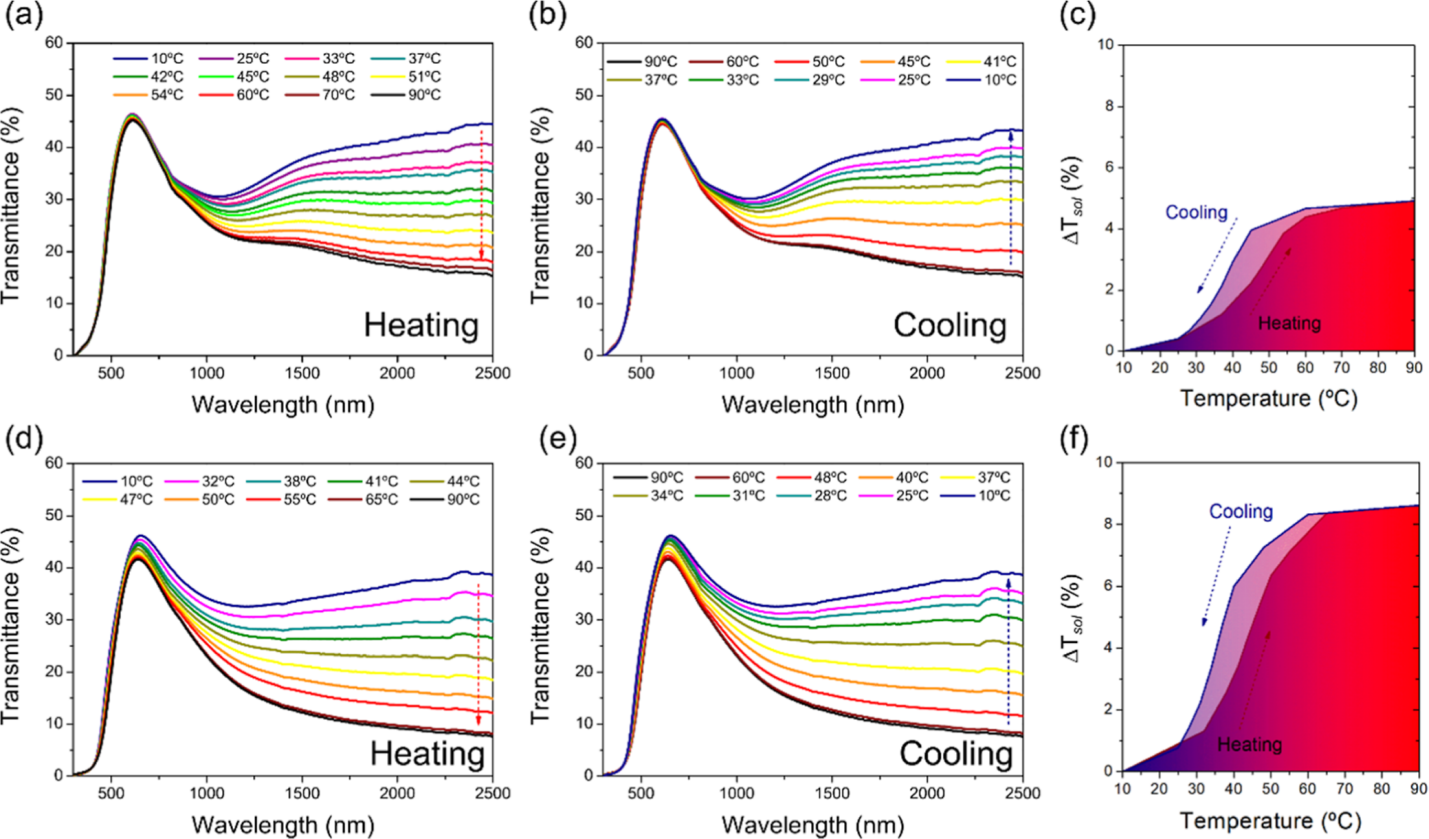
Comparative UV–vis–NIR transmission analysis of samples 6Nb_GL_1 (a–c) and 6Nb_BF_1 (d–f) under sequential thermal cycling. Panels (a,d) show the spectra during the heating phase, while (b,e) show the cooling phase. Panels (c,f) illustrate the corresponding temperature-dependent variation of the Δ*T*_sol_ parameter.

A critical issue that emerges from both the present study and the existing literature is the lack of consensus in the definition of the transition temperature *T*_c_. While the most physically meaningful definition for smart-window operation is the transition temperature during heating, corresponding to the onset of solar blocking, many studies report, sometimes even without explicit clarification, an averaged value between critical temperatures at heating and cooling cycles. This averaged temperature is systematically lower and can be misleading. For instance, in a coating exhibiting a wide hysteresis loop with *T*_c, heat_ = 60 °C and *T*_c, cool_ = 35 °C, the averaged *T*_c_ would be 47.5 °C, despite the fact that the effective solar modulation would not occur until 60 °C. Although the averaged *T*_c_ values have also been included in Table 4 to somehow facilitate comparison with those from previous studies, it should be emphasized that this representation does not accurately reflect the functional behavior of thermochromic coatings in real operating conditions.

In view of these considerations, and with the aim of standardizing and unifying performance metrics for assessing the suitability of VO_2_-based coatings for smart-glazing applications, this work proposes the introduction of a new parameter: the effective transition temperature, *T*_eff_. This temperature is defined as the lowest temperature during the heating cycle at which the coating exhibits a Δ*T*_sol_ exceeding 5%. This threshold is not intended to represent an optimal performance target, but rather a physically meaningful lower bound corresponding to the onset of effective solar modulation. In the literature, VO_2_-based coatings are typically considered technologically competitive when Δ*T*_sol_ approaches ∼10%, together with high luminous transmittance (*T*_lum_ > 60–70%).^8,59^ Within this context, the selected 5% threshold represents a practical criterion to identify the temperature at which the coating begins to exhibit a functionally relevant thermochromic response, while avoiding overly restrictive conditions that could mask the activation behavior of coatings with moderate yet still useful performance. Accordingly, coatings that do not reach this threshold at any temperature can be considered functionally inactive within the explored operating range for smart-window applications.

Under this criterion, the 6Nb coating deposited on soda-lime glass does not qualify as functional, whereas the corresponding coating deposited on borosilicate glass does. Based on the data shown in Figure 10f, the effective transition temperature for the latter is estimated to be 45 °C. This value not only identifies the temperature at which meaningful solar modulation is activated but also confirms that the coating is functionally viable across the entire vis–NIR range, rather than at a single wavelength.

Importantly, *T*_eff_ complements the conventional transition temperature (*T*_c_) by integrating both thermal and optical criteria into a single descriptor, thereby capturing not only when the phase transition occurs but also when it becomes functionally relevant in terms of solar modulation. This distinction is critical, since coatings with similar *T*_c_ values may exhibit markedly different practical performance depending on the magnitude and evolution of Δ*T*_sol_.

Finally, *T*_eff_ is defined in a general manner and can be applied to VO_2_-based coatings regardless of the dopant type, substrate, or film thickness, enabling direct comparison across different studies. Nevertheless, it should not be interpreted as a standalone performance metric but rather in conjunction with *T*_lum_ and maximum Δ*T*_sol_ values. In this sense, *T*_eff_ provides an additional, application-oriented parameter that contributes to a more realistic and standardized assessment of thermochromic coatings for smart-glazing technologies.

## 4 Conclusions

Nb^5+^-doped VO_2_-based thin films were successfully synthesized on soda-lime and borosilicate glass substrates, enabling a systematic assessment of the interplay among dopant concentration, substrate chemistry, and processing conditions. It has been demonstrated that the substrate plays a decisive role on phase stability, microstructural uniformity, and dopant redistribution during thermal oxidation. While VO_2_(M) can be mainly obtained on both substrates, the greater Na diffusion from soda-lime glass promotes the formation of secondary phases and significantly narrows the processing window, whereas borosilicate substrates provide a more stable environment that facilitates enhanced grain coalescence and improved Nb homogenization, as confirmed by electron microscopy.

These differences directly translate into the thermochromic performance of the coatings. Films deposited on borosilicate glass exhibit a more robust and reproducible balance between luminous transmittance and solar modulation efficiency, with reduced sensitivity to dopant concentration and annealing conditions. In contrast, coatings on soda-lime glass experience a clear and progressive degradation of Δ*T*_sol_ at higher Nb contents and reaction temperatures. Kinetic analyses show that Nb effectively reduces the MIT temperature and hysteresis width for both substrates, although multistep transitions are associated with an intrinsic nonuniform Nb incorporation, as evidenced by STEM–EDX and XPS analyses, particularly in soda-lime-supported films.

When compared with W-doped VO_2_ fabricated under similar conditions, Nb requires higher concentrations to achieve comparable reductions in transition temperature but offers a more favorable trade-off between Δ*T*_sol_ and *T*_lum_, with a smaller penalty in solar modulation per dopant atom percentage unit and even improvements in visible transmittance at elevated doping levels. Beyond conventional analysis, the proposed effective transition temperature, *T*_eff_, serves as a physically meaningful and application-oriented descriptor. It establishes a robust framework for evaluating VO_2_-based coatings for smart-glazing technologies, particularly in systems with multistep MIT transitions where a single *T*_c_ fails to accurately characterize thermochromic performance.

## Supplementary Material



## Notes

Section I: extended optical-thermochromic analysis for 14 additional samples not included in the main text; Section II: extended microscopic structural and compositional characterization for samples in Table 2 (PDF)
