# Supplementary material for: Nb-Doped VO2‑Based Coatings on Glass: Substrate Effects, Thermochromic Performance, and an Effective Transition Temperature for Smart-Glazing Applications
Source: ACS Appl Mater Interfaces. 2026 Jun 12;18(24):34462–79. doi: 10.1021/acsami.6c03088 (PMC13307066; doi:10.1021/acsami.6c03088)
Supplement: Supplementary file 1 [file am6c03088_si_001.pdf]

Supporting Information

Nb-doped VO_2-based coatings on glass: Substrate effects, thermochromic performance and an effective transition temperature for smart-glazing applications

Antonio J. Santos[a,b,], Andrea Casas-Acuña[a,b], José M. Mánuel[a,c], Juan J. Jiménez[a,b], Jose M. Obrero-Pérez[a,b], Davide Benedetto[a,b], Nicolas Martin[d], Francisco M. Morales[a,b]*

[a] IMEYMAT: Institute of Research on Electron Microscopy and Materials of the University of Cádiz, E-11510, Puerto Real, Spain.

[b] Department of Materials Science and Metallurgic Engineering, and Inorganic Chemistry, Faculty of Sciences, University of Cádiz, E-11510 Puerto Real, Spain.

[c] Department of Condensed Matter Physics, Faculty of Sciences, University of Cádiz, 11510 Puerto Real, Cádiz, Spain.

[d] SUPMICROTECH, CNRS, Institut FEMTO-ST, 25000 Besançon Cedex, France.

* Corresponding author: antonio.santos@uca.es

Section I. Extended optical-thermochromic analysis for 14 additional samples not included in the main text

Table S1. Deposition and thermal annealing conditions for the additional 14 samples studied. T_r is the reaction temperature (˚C), t_r is the reaction time (s).

**Sample**	**Nb doping (at. %) in $V_xNb_{1-x}O_2$**	**Thickness ratio between $V_{0.94}Nb_{0.06}O_y$ and VO_y**	**Substrate**	**T_r (°C)**	**t_r (s)**
3Nb_GL_S1	1.00	40/40	Glass	550	1
3Nb_GL_S2				550	5
3Nb_BF_S1			Borofloat	525	15
3Nb_BF_S2				550	1
4.5Nb_GL_S1	1.50	60/20	Glass	525	8
4.5Nb_GL_S2					12
4.5Nb_BF_S1			Borofloat	475	120
4.5Nb_BF_S2				525	15
6Nb_GL_S1	2.00	80/0	Glass	475	45
6Nb_BF_S1			Borofloat	450	150
6Nb_BF_S2					240
6Nb_BF_S3				475	90
6Nb_BF_S4				525	15
6Nb_BF_S5				550	5

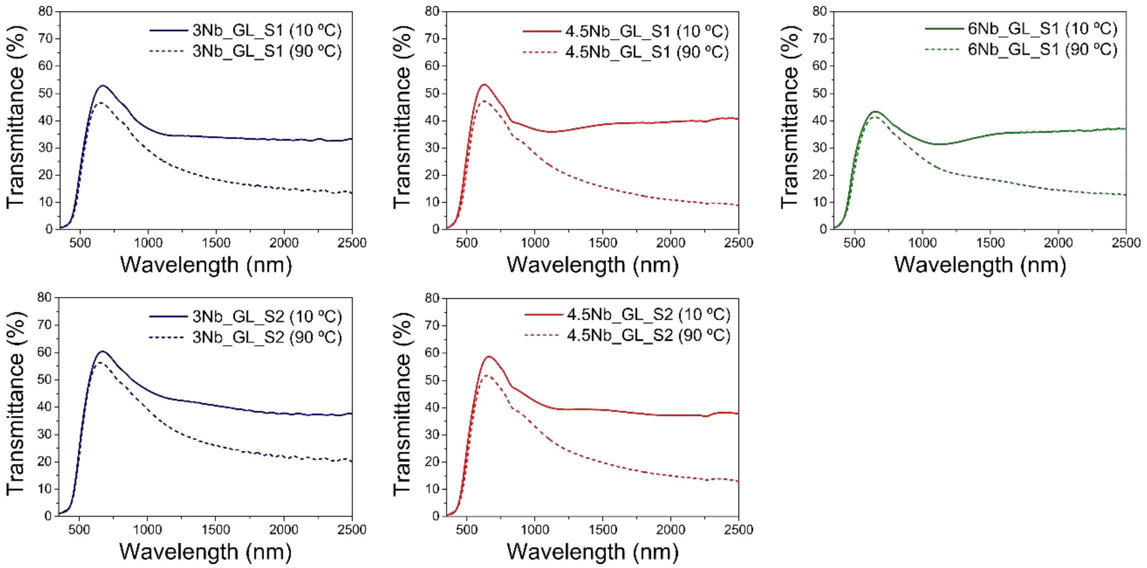

Figure S1. UV-Vis-NIR transmittance spectra for the additional $V_xNb_{1-x}O_2$-based coatings recorded at 10˚C (M1 phase, solid lines) and 90˚C (R phase, dashed lines) on soda-lime glass. Panels are organized by increasing Nb doping: 3Nb (blue), 4.5Nb (red), and 6Nb (green).

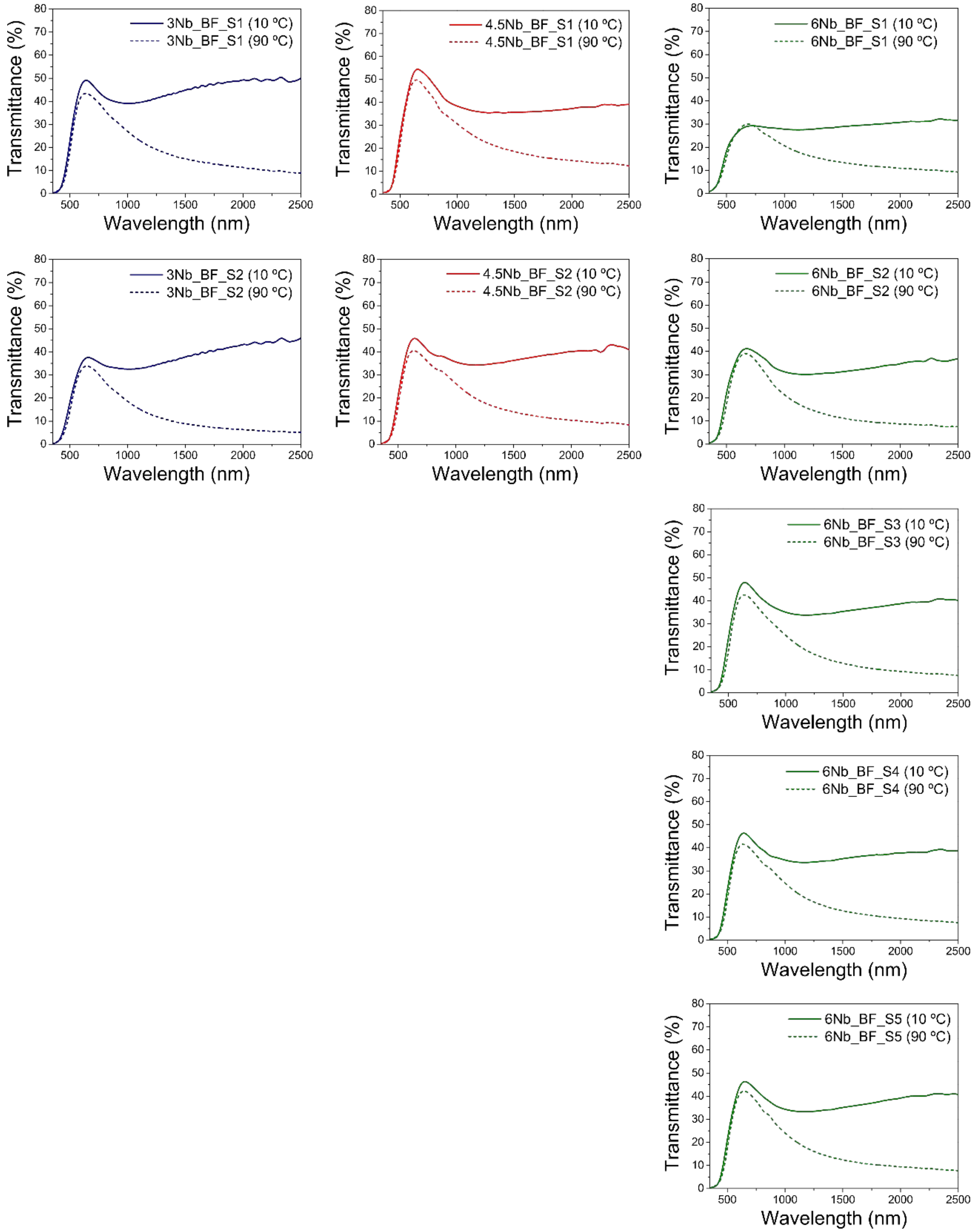

Figure S2. UV-Vis-NIR transmittance spectra for the additional $V_xNb_{1-x}O_2$-based coatings recorded at 10˚C (M1 phase, solid lines) and 90˚C (R phase, dashed lines) on borosilicate glass. Panels are organized by increasing Nb doping: 3Nb (blue), 4.5Nb (red), and 6Nb (green).

Table S2. Radiometric and photometric parameters changes upon heating for the additional 14 samples studied. The accuracy of these values is ±0.1%.

**Sample**	**T_{lum} (%)**	**ΔT_{lum} (%)**	**ΔT_{sol} (%)**
3Nb_GL_S1	36.7	3.3	6.7
3Nb_GL_S2	42.8	1.8	5.4
3Nb_BF_S1	35.9	4.2	9.7
3Nb_BF_S2	27.1	2.7	9.6
4.5Nb_GL_S1	40.8	5.3	8.2
4.5Nb_GL_S2	40.4	4.1	7.9
4.5Nb_BF_S1	39.0	2.6	6.7
4.5Nb_BF_S2	33.1	3.8	8.0
6Nb_GL_S1	34.4	2.2	5.3
6Nb_BF_S1	23.3	0.6	4.3
6Nb_BF_S2	30.4	2.5	7.0
6Nb_BF_S3	34.7	4.5	8.5
6Nb_BF_S4	33.8	3.5	7.9
6Nb_BF_S5	33.3	2.9	7.9

Section II. Extended microscopic structural and compositional characterization for samples in table 2

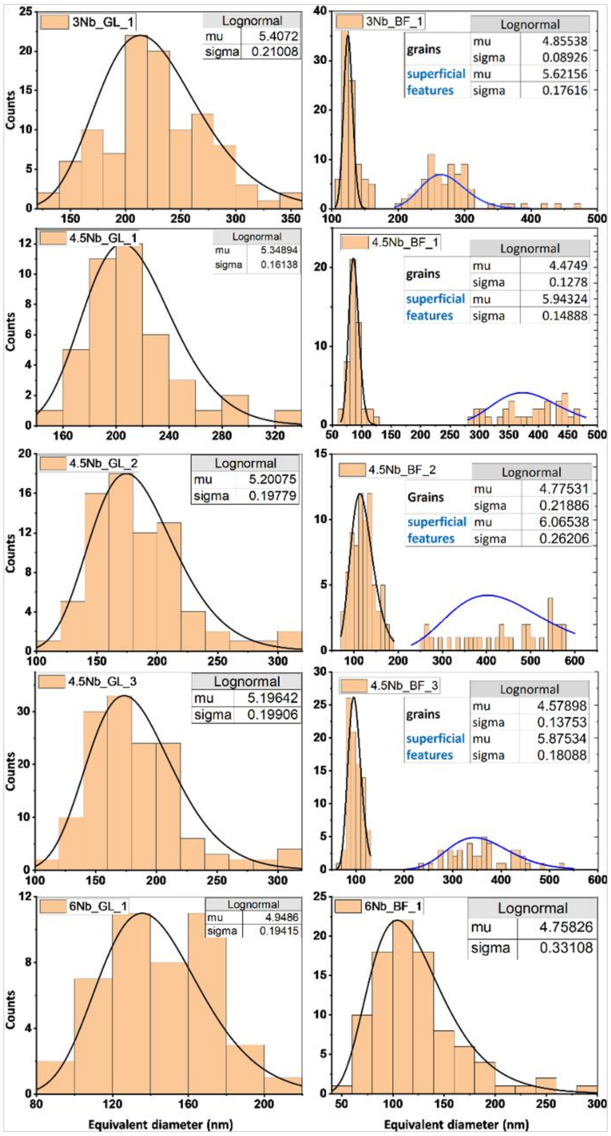

Figure S3. Grain and superficial features size histograms and their fit to lognormal distributions.

The average sizes, $\bar{x}$, and their associated errors, Δx, are calculated, from the μ y σ lognormal-fit parameters using the expressions (Equations S1 and S2):

$$\bar{x} = e^{\left(\mu + \frac{1}{2}\sigma^2\right)} \qquad \text{(S1)}$$

$$\Delta x = \sqrt{e^{2(\mu+\sigma^2)} - e^{(2\mu+\sigma^2)}} \qquad \text{(S2)}$$

Table S3. V and Nb atomic ratio, as percentages (%), measured via EDX at different locations from separated regions of the thin film: (a) region#1: close to the interface (first tens of nanometers); (b) region#2: close to the surface; and (c) the whole thin film (thus, the atomic percentages here correspond to the integrated values of all EDX signals from the complete thin film). The averaged values for measured at regions 1 and 2 are included.

Area \ Sample	4.5Nb_GL_2		4.5Nb_BF_1		6Nb_GL_1		6Nb_BF_1	
	V	Nb	V	Nb	V	Nb	V	Nb
Region#1	96.96	3.04	96.49	3.51	93.84	6.16	92.43	7.57
	96.76	3.24	96.79	3.21	95.09	4.91	93.08	6.92
	96.82	3.18	97.04	2.96	95.51	4.49	93.62	6.38
	96.43	3.57	96.71	3.29	95.99	4.01	94.97	5.03
	96.73	3.27	97.19	2.81	95.42	4.58	93.16	6.84
Averaged value	96.74 ± 0.09	3.26 ± 0.09	96.84 ± 0.12	3.16 ± 0.12	95.17 ± 0.36	4.83 ± 0.36	93.45 ± 0.42	6.55 ± 0.42
Region#2	99.55	0.45	98.37	1.63	96.20	3.80	96.77	3.23
	99.47	0.53	98.59	1.41	96.90	3.10	97.07	2.93
	99.01	0.99	98.10	1.90	97.12	2.88	97.58	2.42
	98.95	1.05	98.69	1.31	97.17	2.83	97.51	2.49
	98.84	1.16	98.05	1.95	95.89	4.11	96.37	3.63
Averaged value	99.16 ± 0.14	0.84 ± 0.14	98.36 ± 0.13	1.64 ± 0.13	96.66 ± 0.26	3.34 ± 0.26	97.06 ± 0.23	2.94 ± 0.23
Whole film	97.34 ±11.94	2.66 ± 0.43	97.19 ±11.91	2.81 ± 0.45	96.01 ± 0.53	3.99 ± 0.53	95.92 ± 0.54	4.08 ± 0.54

In order to know more about the effect of the initial layer architecture upon the behavior of niobium during its thermal treatment, additional FIB lamellae of samples 4.5Nb_GL_2 and 4.5Nb_BF_1 were prepared and inspected by STEM. The main conclusions of these experiments are compiled in Fig. S4. First of all, the HAADF micrographs of these samples (Fig. S4(a) and (d)) allowed to identify two distinct $V_xNb_{1-x}O_y$ regions or sublayers, especially after combining these images with the net counts maps of either Nb (Fig. S4(b) and (f)) or V (Fig. 43(c) and (e)). In order to ease the

comparison with the 6Nb samples, the average composition of each film as a whole is given in each HAADF micrograph. One can conclude from these maps that Nb is indeed mostly present at the bottom region of the annealed structure, whereas the top half is much poorer in this element. Furthermore, there are some Nb-rich accumulations that suggest chemical heterogeneities comparable to the ones in the 6Nb samples. It is worth remarking that the sudden increase of Nb net counts beyond the actual $V_xNb_{1-x}O_y$ film is a false positive signal caused by Pt-M and Nb-L X-ray lines overlapping, so it must be ignored. Apart from this, these results agree with what was found in the single-layer samples in the sense that Nb incorporation is heterogeneous. According to the EELS mapping experiments successfully carried out at the bottom region, it is again formed by VO_2. In both integrated spectra, shown in Fig. S4 (g) and (h) for samples 4.5Nb_GL_2 and 4.5Nb_BF_1 respectively, the same four peaks are found at roughly the same energy loss values, confirming the same oxidation state for vanadium.

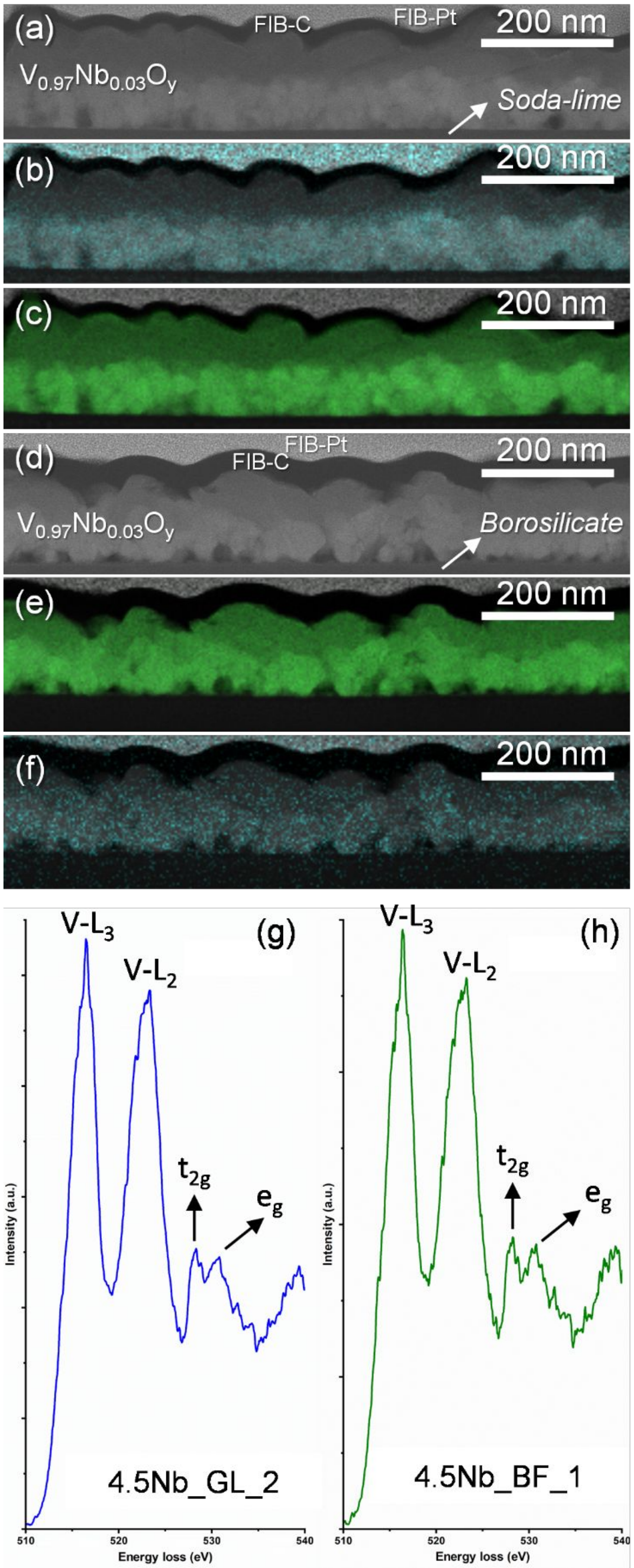

(a)
FIB-C
FIB-Pt
200 nm
$V_{0.97}Nb_{0.03}O_y$
Soda-lime
(b)
200 nm
(c)
200 nm
(d)
FIB-C
FIB-Pt
200 nm
$V_{0.97}Nb_{0.03}O_y$
Borosilicate
(e)
200 nm
(f)
200 nm
V-L3
V-L2
(g)
t2g
eg
Intensity (a.u.)
4.5Nb_GL_2
Energy loss (eV)
V-L3
V-L2
(h)
t2g
eg
Intensity (a.u.)
4.5Nb_BF_1
Energy loss (eV)

Figure S4. For samples 4.5Nb_GL_2 and 4.5Nb_BF_1, respectively: STEM-HAADF micrographs (a, d); EDX net counts maps of Nb (b, d) and V (c, e) added over the micrographs; and integrated EELS spectra retrieved from the first $V_xNb_{1-x}O_y$ sublayer (g, h).

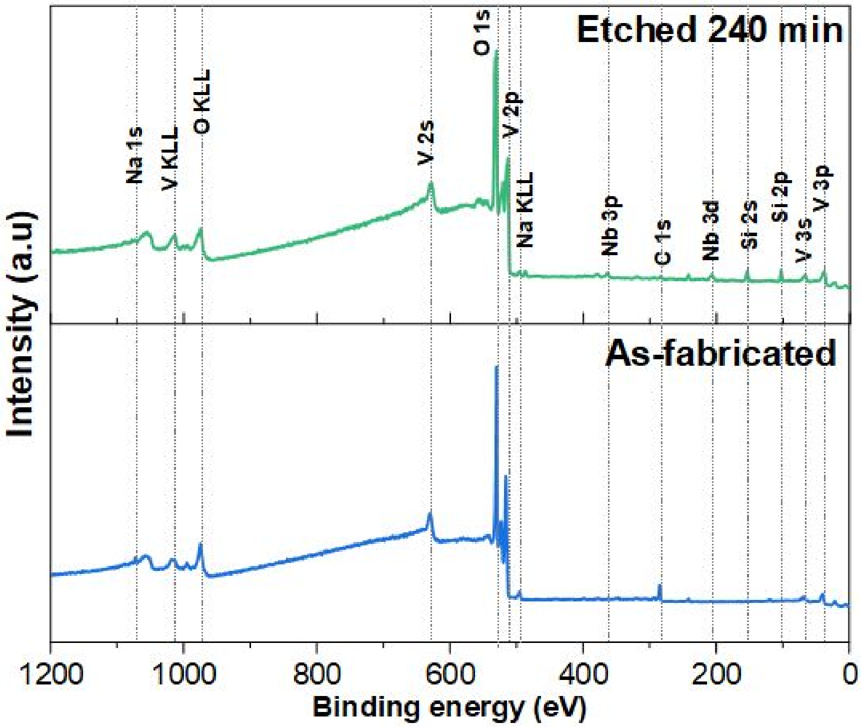

Figure S5. XPS survey spectra of the 6Nb-VO_2 coating deposited on a borosilicate glass substrate before (bottom) and after (top) Ar^+ ion etching.
